# Asymmetric oligomerization state and sequence patterning can tune multiphase condensate miscibility

**DOI:** 10.1038/s41557-024-01456-6

**Published:** 2024-02-21

**Authors:** Ushnish Rana, Ke Xu, Amal Narayanan, Mackenzie T. Walls, Athanassios Z. Panagiotopoulos, José L. Avalos, Clifford P. Brangwynne

**Affiliations:** 1 Department of Chemical and Biological Engineering, Princeton University, Princeton, NJ USA; 2 Howard Hughes Medical Institute, Princeton University, Princeton, NJ USA; 3 Andlinger Center for Energy and the Environment, Princeton University, Princeton, NJ USA; 4 Omenn-Darling Bioengineering Institute, Princeton University, Princeton, NJ USA

**Keywords:** Intrinsically disordered proteins, Biopolymers in vivo, Computational models

## Abstract

Endogenous biomolecular condensates, composed of a multitude of proteins and RNAs, can organize into multiphasic structures with compositionally distinct phases. This multiphasic organization is generally understood to be critical for facilitating their proper biological function. However, the biophysical principles driving multiphase formation are not completely understood. Here we use in vivo condensate reconstitution experiments and coarse-grained molecular simulations to investigate how oligomerization and sequence interactions modulate multiphase organization in biomolecular condensates. We demonstrate that increasing the oligomerization state of an intrinsically disordered protein results in enhanced immiscibility and multiphase formation. Interestingly, we find that oligomerization tunes the miscibility of intrinsically disordered proteins in an asymmetric manner, with the effect being more pronounced when the intrinsically disordered protein, exhibiting stronger homotypic interactions, is oligomerized. Our findings suggest that oligomerization is a flexible biophysical mechanism that cells can exploit to tune the internal organization of biomolecular condensates and their associated biological functions.

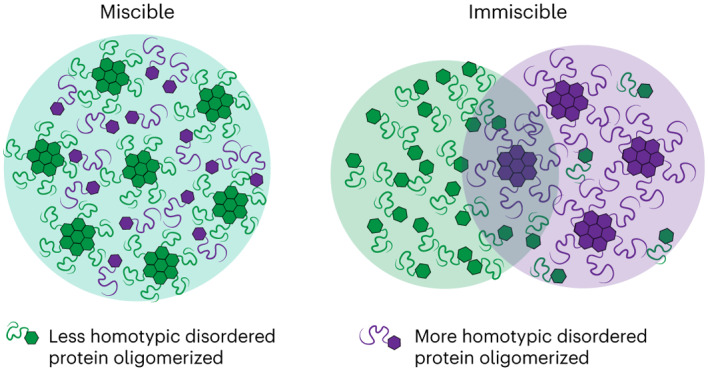

## Abstract (web summary)

The biomolecular principles underlying the formation of multiphasic condensates have been difficult to elucidate owing to a paucity of tools, especially within living cells. In this work synthetic orthogonal protein scaffolds alongside molecular simulations are used to highlight how the oligomerization of disordered proteins can asymmetrically drive miscibility–immiscibility transitions.

## Main

Many cellular reactions are facilitated by the colocalization of specific sets of biomolecules into different intracellular organelles such as mitochondria, Golgi apparatus and endoplasmic reticulum. In addition to the classic membrane-bound organelles (for example, mitochondria, Golgi apparatus and endoplasmic reticulum), there are a variety of membraneless organelles (for example, stress granules, P-bodies and nucleoli) that support diverse cellular functions, from the sequestration of translationally stalled messenger RNA (mRNA) to ribosome biogenesis. Over the last decade, a large number of studies have established biomolecular liquid–liquid phase separation (LLPS) and related phase transitions as a mechanism for the assembly of these structures, which are typically referred to as biomolecular condensates^1–4^. These findings provide a framework that brings fundamental concepts in thermodynamics and polymer physics to bear on a key aspect of intracellular organization^5–9^.

Past work has shown that the classical Flory–Huggins framework of polymer phase separation is capable of capturing key features of intracellular LLPS^5,10–12^. Above the saturation concentration, the free energy change of dispersed biomolecules condensing into droplets outweighs the entropic loss. The Flory–Huggins framework has been helpful for conceptualizing how molecular components of condensates, particularly proteins and nucleic acids with an extended and high multivalent interaction capacity, can impact this interplay between molecular interactions and mixing entropy to promote phase separation. For example, proteins enriched in condensates often exhibit substantial intrinsically disordered protein regions (IDRs), which together with nucleic acid binding partners have been established as key drivers of condensate formation^13–15^. Such multivalent biomolecular components can be represented as coarse-grained polymers composed of ‘sticker’ regions interspersed with inert spacers^16,17^. These ‘stickers’ are understood to enable relatively short-ranged favourable interactions and have been recently characterized in purified proteins^10^.

Despite the utility of the Flory–Huggins framework, living cells are far more complex than the inanimate systems it was originally formulated to describe. For instance, cells can dynamically modulate both the contact interactions and entropic factors for regulating the formation of condensates, for example, through post-translational modifications such as phosphorylation and methylation that tune interactions between sticky regions^18,19^. Living cells also make extensive use of the dynamic oligomerization of biomolecular components to modulate LLPS^20–22^. Oligomerization can reduce the entropic cost of LLPS by increasing the effective chain length of proteins, thereby functioning as an entropic knob. Alternatively, monomers can also be potentially sequestered away into stable oligomers, which could hinder LLPS^23^. Oligomerization has also been harnessed to allow the formation of de novo condensates^24–27^. In one such system, optogenetic Corelet technology, the light-dependent oligomerization of IDRs and other proteins, enabled the quantitative mapping of phase diagrams in living cells and has been used to probe the biophysical properties of condensate nucleation and coarsening behaviour^28,29^.

These and other studies demonstrate that although cells are highly complex, fundamental concepts from polymer physics and thermodynamics can be fruitfully employed to understand and engineer intracellular phase behaviour. Indeed, dynamic protein oligomerization plays a central role in driving the formation of condensates such as nucleoli^30,31^, nuclear speckles^32^ and stress granules and P-bodies^22^. Interestingly, these and many other condensates are also often multiphasic, with compositionally distinct phases that are thought to be relevant for their biological functions. For example, the nucleolus is organized into a ‘core–shell’ structure where transcription of ribosomal RNA (rRNA) occurs at the inner ‘core’ (that is, the fibrillar centre and dense fibrillar component region), and the nascent rRNA transcripts then undergo sequential maturation steps in the surrounding fluid ‘shell’ (that is, granular component region) before fluxing out of the nucleolus^21,31^. Nuclear speckles also exhibit such a core–shell architecture, while stress granules, P-bodies and other condensates provide more complex examples of multiphase organization throughout the cell^33^.

Like most condensates, these archetypal multiphase condensates typically harbour proteins with a notable fraction of IDRs. However, the role of IDRs in driving multiphase organization is not well understood. Recent studies provide indirect evidence that IDRs by themselves might be insufficient to provide specificity for nucleating multiple coexisting phases^22,30,34^, and the molecular mechanisms underlying multiphase formation are poorly understood, particularly within the context of living cells. However, investigating potential mechanisms has proven to be challenging due to the inherent complexity of the intracellular milieu and a lack of tools for probing phase immiscibility in vivo. As a result, how biomolecular components encode their own organization into multiple immiscible subcompartments in living cells remains unclear.

Here we combine intracellular reconstitution experiments in mammalian and yeast cell systems with coarse-grained molecular simulations to demonstrate that while IDR sequence patterning can by itself dictate multiphase organization, oligomerization greatly amplifies the tendency for segregation into distinct condensed phases. Furthermore, we find that the effect of oligomerization in driving condensate immiscibility is ‘asymmetric’, with stronger homotypic IDRs showing greater immiscibility when differentially oligomerized. We propose that fine-tuning the oligomerization state of proteins is a mechanism by which cells can modulate the multiphasic organization of biomolecular condensates when the differences in IDR sequence patterning are otherwise insufficiently strong to drive immiscibility.

## Results

### Role of oligomerization in driving immiscibility

**Fig. 1:**
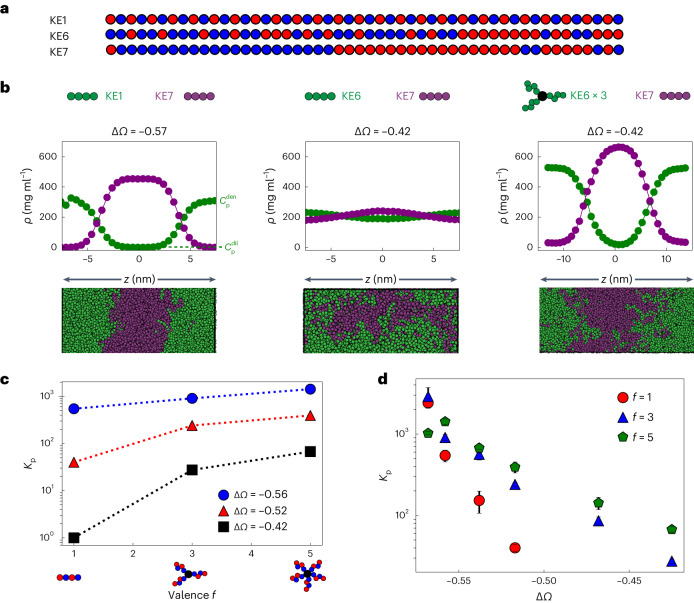
The effect of oligomerization in driving multiphase immiscibility. **a**, Model polyampholyte sequences used (Extended Data Table 1). Red beads represent negatively charged glutamate (E) residues while blue beads represent positively charged lysine residues (K). **b**, Simulated density (*ρ*) profiles and snapshots of binary mixtures of model polyampholytes highlight the dependence of multiphase immiscibility on the SCD difference (Δ*Ω*) and oligomerization. For the snapshots, in all cases KE7 was coloured dark magenta while the other sequence was coloured green for visualization; *z* is a distance. **c**, Dependence of partitioning on the oligomerization state for three different binary polyampholyte pairs. Partitioning *K*_p_ was estimated as the ratio of the concentration of component p in the ‘q-lean’ phase to the concentration of p in the ‘q-rich’ phase. In all cases, q was chosen as KE7. **d**, Variation of the partitioning with charge patterning across different oligomerization states, highlighting how increased oligomerization enhances the range across which polyampholyte pairs can remain immiscible. Error bars represent ± the standard error of the mean for partitioning obtained over independent simulation frames. Source data

To study condensate miscibility, we first used coarse-grained implicit solvent molecular dynamics simulations to examine how oligomerization can drive phase separation. IDRs were modelled as charge-neutral ‘KE’ polyampholytes, that is, polymers comprising a string of either positively (K) or negatively (E) charged beads (Fig. 1a). Building from prior studies^35^, we used a set of polyampholytes (KE1–KE7; Extended Data Table 1), representing sequences of increasing charge ‘blockiness’, quantified by the sequence charge decoration (SCD) metric^36,37^. Consistent with previous studies^34,38^, we find that chains that exhibit major differences in their patterning form two compositionally distinct phases when mixed (for example, KE1 and KE7; Fig. 1b).

**Extended Data Fig. 1:**
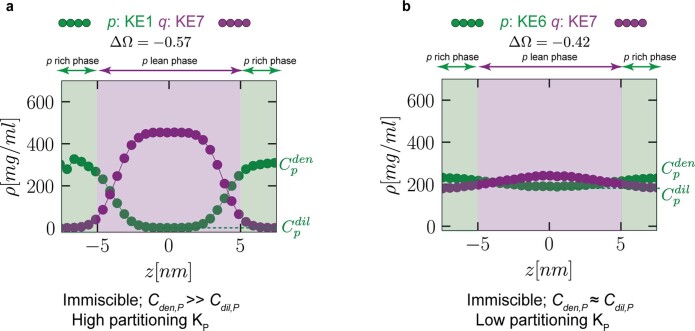
Partitioning metric as a proxy for immiscibility. **a**. Density profiles of immiscible KE1 and KE7 showing the extent of the two phases and the high partitioning. **b**. Density profiles of relatively miscible KE6 and KE7 showing marginal demixing between the two phases and the low partitioning. Source data

To simplify the analysis of the sequence dependence of this immiscibility, we varied the charge patterning of one of the components p while keeping the second component q (for example, KE7) fixed. Furthermore, since two immiscible phases, by definition, will each largely exclude the partitioning of the unfavourable component, we quantify miscibility by examining the partitioning of the component p in the phase of component q; since component q is fixed (KE7), this mapping is consistent with other immiscibility metrics used in the literature and our own analysis (Extended Data Fig. 1), showing partitioning is a good proxy for phase miscibility. We define the partitioning metric as ${K}_{{\mathrm{p}}}=\frac{{C}_{{\mathrm{p}}}^{{{\mathrm{den}}}}}{{C}_{{\mathrm{p}}}^{{{\mathrm{dil}}}}}$, where ${{C}}_{{\mathrm{p}}}^{{{\mathrm{den}}}}$ is the concentration of component p in the ‘q-lean’ phase, while ${C}_{{\mathrm{p}}}^{{{\mathrm{dil}}}}$ is the concentration in the ‘q*-*rich’ phase. We find the degree of miscibility is dependent on the difference in charge patterning, with larger differences in charge patterning leading to greater immiscibility (for example, in Fig. 1b, compare KE1 and KE7 versus KE6 and KE7).

In examining how different the sequences must be to drive changes in partitioning/miscibility, we found an unexpectedly strong sequence dependence. For example, reducing the difference in charge patterning −Δ*Ω*, a normalized form of the SCD metric^39^, by only 36% (Fig. 1b) caused the partitioning to decrease by two orders of magnitude. Indeed, our findings suggest that large differences in sequence patterning, and therefore effective interaction strengths, are required for IDRs to form distinct phases. Conversely, IDRs without such differences in charge or sequence patterning might be incapable of demixing into immiscible phases. Thus, the specificity of IDR–IDR interactions alone might, in practice, be insufficient for generating multiple immiscible phases and instead require additional mechanisms to amplify specificity.

Recent work using synthetic scaffolds has shown that oligomerization can greatly increase the propensity for phase separation by reducing the entropic cost of demixing^2,40^. This can be understood within the Flory–Huggins framework, where condensation is a result of the competition between the entropic cost of mixing and energy gain due to attractive contact interactions. Increasing the oligomerization state leads to a reduction in the entropic cost, thereby enhancing phase condensation^5^. We hypothesized that a similar mechanism might be used also to drive segregation into multiple distinct condensed phases. To examine the role that oligomerization might play in amplifying immiscibility, we simulated binary mixtures where component p was modelled as a star polymer with *f* number of arms while component q was kept as a single chain. To ensure equal stoichiometry, the mass fraction of the two components was kept equal and two different oligomerization states of *f* = 3 and 5 were considered. For all cases, we find that increased oligomerization leads to enhanced immiscibility; this can be observed clearly in the case of KE6/KE7 mixtures, which largely intermingle with little or no distinct phases apparent, while trimerization of KE6 (KE6 × 3/KE7) gives rise to two distinct phases (Fig. 1b). Immiscibility, as quantified by the fold change in the partitioning, reveals a dependence on both the valence and the difference in charge patterning, with more miscible pairs of IDRs having a greater fold change in partitioning upon adding valence (Fig. 1c). Simulating a range of binary sequence pairs (Extended Data Table 2), we find that higher oligomerization states enable multiphase formation by sequences with relatively similar charge patterning, suggesting that oligomerization effectively amplifies the importance of sequence-encoded interaction preferences (Fig. 1d).

### Oligomerization can drive demixing of exogenous proteins

**Fig. 2:**
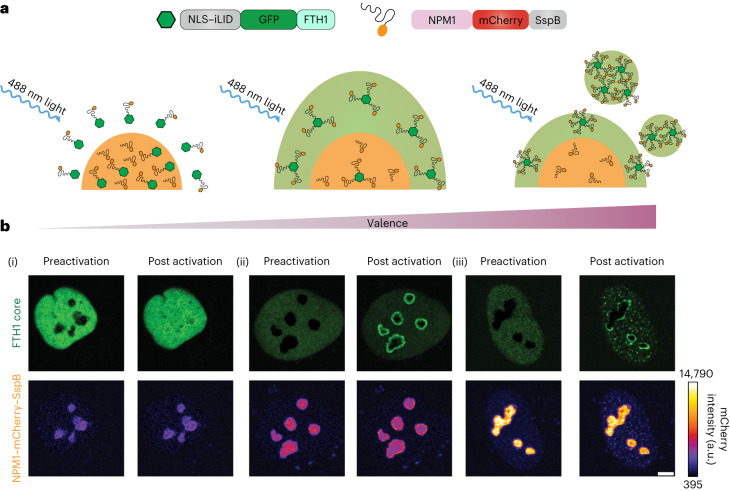
Light-induced oligomerization drives demixing of nucleolar localized proteins. **a**, Schematics of the optogenetic Corelet experiment in living cells showing oligomerization-driven demixing from the nucleolus. At low valence, the NPM1–mCherry–SspB remains within the nucleolus (orange phase). As valence is increased, there is first an interfacial enrichment of NPM1, followed by formation of de novo droplets (green phase). Higher valence implies a greater fraction of NPM1–mCherry–SspB is bound to the ferritin core. **b**, Representative images of the valence-dependent changes in nucleolar distribution of NPM1 before and after blue-light activation in the nucleus of live U2OS cells. Before light activation, the ferritin core is diffuse throughout the nucleoplasm while the NPM1–mCherry–SspB is strongly partitioned into the nucleolus. Light-induced oligomerization of NPM1–mCherry–SspB was seen to change its localization. The scale bar represents 5 μm. Images shown are representative of *n* = 5 independent photoactivation experiments for NPM1–mCherry–SspB. Source data

To probe the oligomerization-driven demixing hypothesis within living cells, we used the light-inducible Corelet system^24,40^, which enables light-triggered dimerization of SspB–iLID (SspB, stringent starvation protein B; iLID, the iLID heterodimer with Protein Data Bank no. 4WF0) and oligomerization of intracellular proteins or IDRs. We co-expressed nucleophosmin-1 (NPM1) fused to SspB (NPM1–mCherry–SspB), together with iLID fused to a multivalent (24-mer ferritin, FTH1) core and a nuclear localization signal (NLS–iLID–GFP–FTH1; GFP, green fluorescent protein; Fig. 2a), in human osteosarcoma (U2OS) cells. Upon blue-light-induced SspB–iLID dimerization, NPM1 binds to the 24-mer ferritin core, thereby increasing its valence. Without exogenous oligomerization, the NPM1 construct is strongly partitioned into the nucleolus (Fig. 2b, valence = 0.7). However, upon blue-light activation we observe a striking valence-dependent demixing response. At low overall valence (that is, ratio of NPM1 concentration to core concentration), we see that the localization of NPM1 remains almost unchanged (Fig. 2b, valence = 0.7). However, for cells with higher valence, the NPM1 exhibits a marked demixing from the nucleolus and instead becomes enriched at the nucleolar periphery (Fig. 2b, valence = 4.3). Interestingly, at the very highest valence, NPM1 can both demix to the nucleolar periphery and form new separate condensates in the nucleoplasm (Fig. 2b, valence = 34). Taken together, both our simulation and experimental results suggest that oligomerization can cause demixing of condensate components, driving them to form new condensates or multiphase structures demixed from existing condensates.

### IDR-core proteins probe condensate immiscibility in vivo

**Fig. 3:**
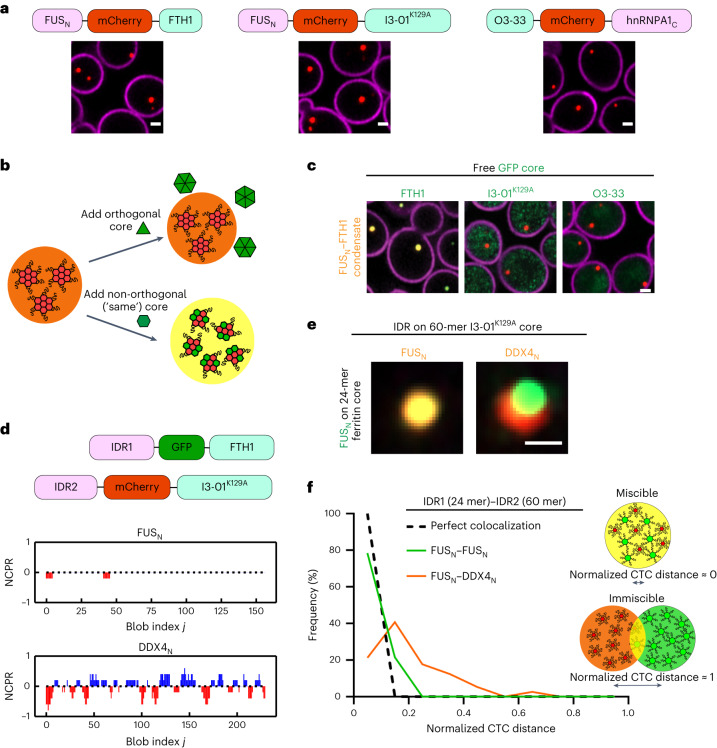
Synthetic and orthogonal IDR-core proteins to study phase immiscibility. **a**, Representative images of constitutive condensates formed by synthetic IDR-core proteins in yeast (the source data files contain details). Scale bars represent 1 μm. **b**, Schematic diagram of the experiment to examine the orthogonality among different cores (FTH1, I3-01^K129A^ and O3-33). Condensate formed by one core should not recruit other cores if the cores are orthogonal. **c**, Only GFP-tagged free ferritin cores are recruited to FUS_N_–FTH1 condensate (I3-01^K129A^ and O3-33 cores are not recruited). The scale bar represents 1 μm. Images shown here are representative of the experiment with *n* = 3 replicates performed for each condition (the source data files contain details). **d**, Schematics of the orthogonal IDR-core systems; the residue charge distributions of FUS_N_ and DDX4_N_ are quantified using the net charge per residue (NCPR). The 24-mer FTH1 and the 60-mer I3-01^K129A^ cores are used for studying the interactions between condensate phases formed by different IDRs, for example, FUS_N_ and DDX4_N_. **e**, Representative images of FUS_N_–FTH1 condensate interacting with FUS_N_– or DDX4_N_–I3-01^K129A^ condensate. The scale bar represents 0.5 μm. **f**, The frequency distribution of the normalized CTC distance between condensates (FUS_N_–FUS_N_, *n* = 93; FUS_N_–DDX4_N_, *n* = 113). Normalization is obtained by dividing the measured distance between condensates with the sum of the radii of the two condensates. The ‘perfect colocalization’ is plotted by setting the frequency to 100% for the lowest bin (black dashed line; Methods for details). The normalized CTC distance is approximately equal to 0 for two miscible condensates and is approximately equal to 1 for two associated immiscible condensates. The ‘IDR1 (24 mer)–IDR2 (60 mer)’ represents respective fusion of the two IDRs to the cores. For example, FUS_N_–DDX4_N_ indicates the FUS_N_ is fused to 24-mer ferritin core and DDX4_N_ is fused to 60-mer I3-01^K129A^ core. All measurements are independent. Source data

The preceding NPM1 data are consistent with our simulations, together showing how condensate components can demix into multiphase condensates upon oligomerization. However, the nucleolus is a highly complex and multicomponent structure that itself natively exhibits multiphase organization^30^, complicating the elucidation of the underlying physics. We thus designed a more tractable intracellular system that allows for the reconstitution of multiple distinct synthetic condensates in *Saccharomyces cerevisiae*. This approach builds from the Corelet technology (Fig. 2)^40^, but here we use a constitutive oligomerization approach (that is, not light dependent) so that synthetic biomolecular condensates are always present in cells. Three different cores are used: in addition to the previously described ferritin protein composed of 24 human ferritin heavy chain subunits (FTH1), we also use a 60-mer synthetic protein I3-01^K129A^ (ref. ^41^) and a 24-mer synthetic protein O3-33 (ref. ^42^). Upon expression in *S. cerevisiae*, mCherry-tagged cores fused to IDRs, such as the N-terminal FUS IDR (FUS_N_) or the C-terminal IDR of heterogeneous nuclear ribonucleoprotein A1 (hnRNPA1_C_), drive the formation of constitutive intracellular condensates (Fig. 3a). This is consistent with previous findings that oligomerization drives the intracellular LLPS of various IDRs including FUS_N_ and hnRNPA1_C_ (refs. ^40,43,44^).

**Extended Data Fig. 2:**
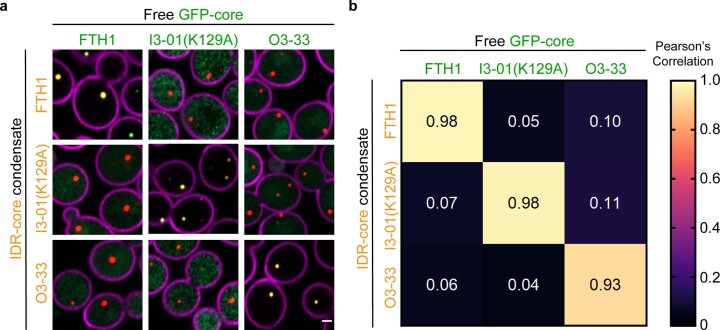
Additional data demonstrate core orthogonality related to Fig. 2. **a**. Representative confocal fluorescence microscope images of yeast expressing combinations of mCherry-tagged IDR-core condensate and GFP-tagged free core (See source data files for details). Scale bars, 0.5 μm. **b**. The degree of recruitment of free core into the condensate as measured using Pearson’s correlation. Source data

For these different oligomerizing cores to be useful in examining the biophysical determinants of multiphase immiscibility, they must be orthogonal; that is, each core unit must be capable of self-assembly, independent of the other one. To test for such orthogonality, we expressed GFP-tagged free core subunits (that is, without any IDRs) in the presence of a different core fused to IDRs; if the cores are orthogonal, then we expect the IDR-core to form a condensate that does not contain the other core (Fig. 3b). Consistent with orthogonality, we find that IDR-core condensates recruit only subunits of the same core, and when the free core is different from the condensate core, the GFP-tagged core subunits are distributed throughout the yeast cell (Fig. 3c and Extended Data Fig. 2).

We proceeded to test whether these orthogonal IDR-core systems can be used to study condensate immiscibility by using two IDRs (FUS_N_ and DDX4_N_) that have distinct driving forces for phase separation (Fig. 3d). The FUS_N_ prion-like domain contains evenly distributed aromatic residues that are known to be important for its phase separation^45–47^, while DDX4_N_ phase separation is primarily mediated by electrostatic interactions^13,15,48^. We find that FUS_N_–FTH1 condensates are not fully miscible with DDX4_N_–I3-01^K129A^ condensates (Fig. 3e), a result that is consistent with recent findings in vitro^48^. However, despite their immiscibility, FUS_N_ and DDX4_N_ condensates tend to associate, suggesting some favourable mutual interactions that lead to a reduced surface tension between the two condensate phases. To quantify the degree of miscibility between condensate phases, we measured the centre-to-centre (CTC) distance between overlapping condensates normalized by the sum of their radii. As expected, the normalized CTC distance profile of the immiscible FUS_N_ and DDX4_N_ pair is markedly different from the profile of a FUS_N_–FUS_N_ control (Fig. 3f). Taken together, our data show that these orthogonal IDR-core systems can be used to study IDR-driven condensate immiscibility and that a significant difference in the sequences of oligomerized IDRs can give rise to condensate immiscibility.

### Oligomerization can drive the immiscibility of condensates

In the preceding in vivo experiments, we demonstrated that our engineered orthogonal IDR-core proteins can be used for studying the condensate phase immiscibility. To further elucidate the role of oligomerization in tuning condensate immiscibility when the IDRs are not drastically different in their sequence patterning, we sought to use IDRs that share similar driving forces for phase separation. We turned to the C-terminal low-complexity domain of heterogeneous nuclear ribonucleoprotein A1 (hnRNPA1_C_), whose phase separation is driven by aromatic residues similar to FUS_N_ (refs. ^10,49^).

**Fig. 4:**
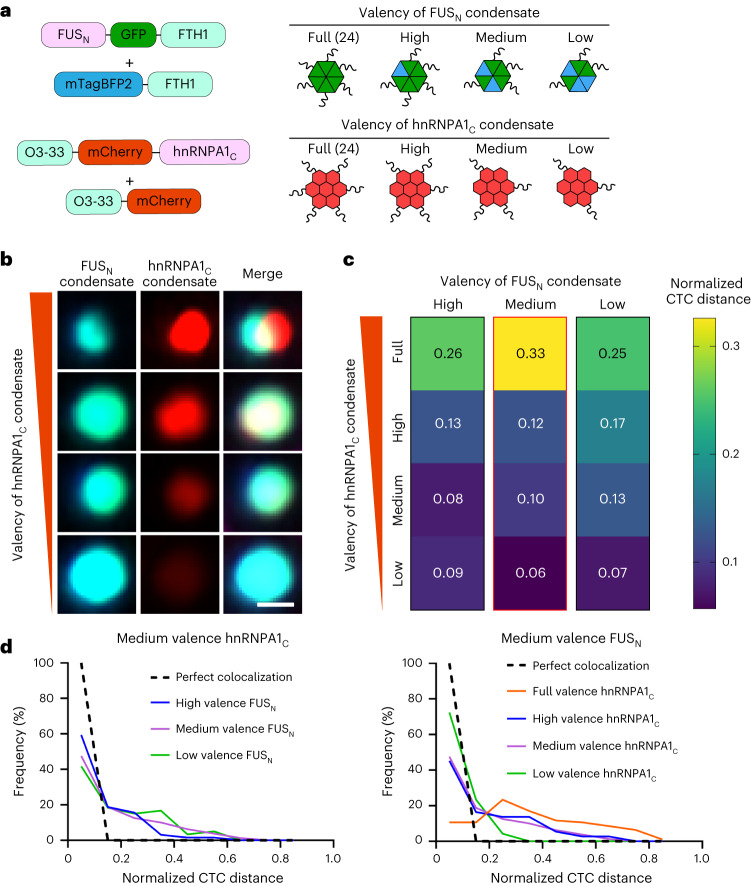
Oligomerization can drive the miscibility–immiscibility transition of synthetic IDR condensates in vivo. **a**, Schematics of the FUS_N_–FTH1 and hnRNPA1_C_–O3-33 condensates. Both ferritin and O3-33 are 24-mer cores. mTagBFP2 is a blue fluorescent protein. The valence can be lowered by expressing fluorescent-protein-tagged free cores without the IDRs. **b**, Example images of the miscibility–immiscibility transition by varying the valence of hnRNPA1_C_ condensate while keeping the valence of FUS_N_ condensate fixed. The scale bar represents 0.5 μm. **c**, The medians of the normalized CTC distance between FUS_N_ and hnRNPA1_C_ condensates at a range of valence values (*n* > 36). All measurements are independent (source data files contain details). The representative images shown in **b** are from the column outlined in red. **d**, Examples of changes in frequency distribution of the normalized CTC distance between FUS_N_ and hnRNPA1_C_ condensates when the valence of one IDR is fixed, and the other IDR varies. Source data

**Extended Data Fig. 3:**
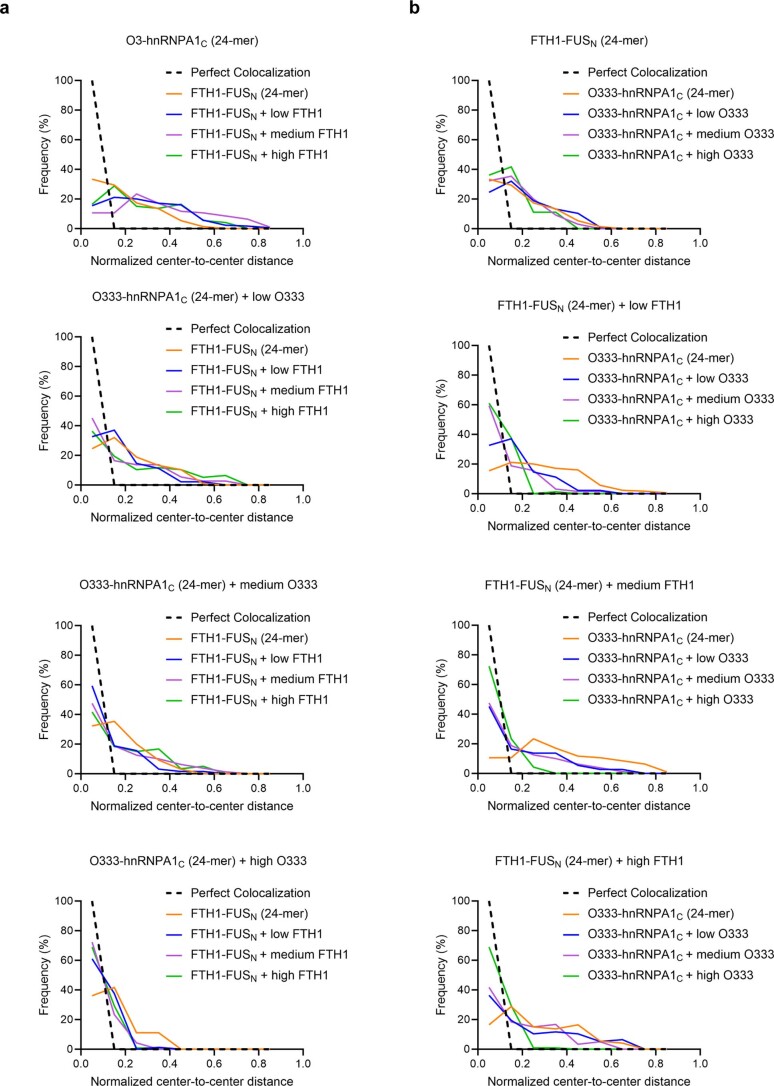
The normalized center-to-center distribution between FUS_N_-FTH1 and hnRNPA1_C_-O3-33 condensates Related to Fig. 3. **a**. Varying FUS_N_ valence at a fixed hnRNPA1_C_ valence. **b**. Varying hnRNPA1_C_ valence at a fixed FUS_N_ valence. Source data

We again used our orthogonal IDR-core systems, in which the 24-mer ferritin and 24-mer O3-33 cores were used to oligomerize FUS_N_ and hnRNPA1_C_, respectively. To obtain varying valences of IDRs, we expressed free ferritin and O3-33 core subunits in the presence of 24-mer FUS_N_–ferritin and 24-mer hnRNPA1_C_–O3-33 condensates (Fig. 4a). Specifically, additional gene copies of ferritin were expressed to reduce the effective valence of FUS_N_, and a set of yeast promoters with well-characterized expression strength (strong *TDH3p*, medium *HHF2p* and weak *RPL18Bp*) was used to overexpress O3-33 core subunits to reduce the effective valence of hnRNPA1_C_ (ref. ^50^). When the valences of FUS_N_ and hnRNPA1_C_ are both high, we observe two immiscible condensates, a FUS_N_-rich phase and an hnRNPA1_C_-rich phase (Fig. 4b,c). Interestingly, when the valence of the FUS_N_ condensate is fixed and the valence of the hnRNPA1_C_ condensate is lowered from 24, we observed that the two condensate dense phases become miscible (Fig. 4b–d and Extended Data Fig. 3). By contrast, we find that for a fixed hnRNPA1_C_ valence, lowering the FUS_N_ condensate valence does not lead to significant change in miscibility (Fig. 4c,d and Extended Data Fig. 3). Taken together, these findings suggest that condensate miscibility can be tuned by the oligomerization state of its protein constituents and, in this case, the oligomerization state of hnRNPA1_C_ is more critical to the miscibility of these two condensates than the oligomerization state of FUS_N_.

### Oligomerization asymmetrically tunes condensate immiscibility

**Fig. 5:**
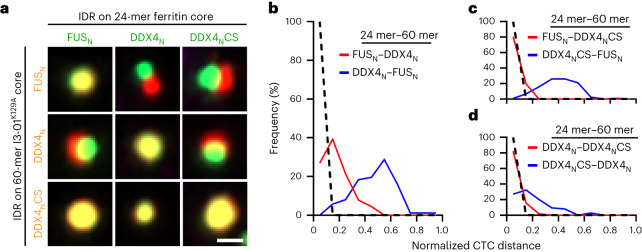
The interplay of oligomerization and IDR sequence patterning in modulating immiscibility in vivo. **a**, Examples of images of miscible and immiscible condensate phases formed by combination of cores (ferritin and I3-01^K129A^) and IDRs (FUS_N_, DDX4_N_ and DDX4_N_CS). The scale bar represents 0.5 μm. **b–d**, Changes in frequency distribution of the normalized CTC distance of IDR–IDR pairs when the specific oligomerization state is interchanged. FUS_N_–DDX4_N_, *n* = 89; DDX4_N_–FUS_N_, *n* = 87 (**b**). FUS_N_–DDX4_N_CS, *n* = 107; DDX4_N_CS–FUS_N_, *n* = 119 (**c**). DDX4_N_–DDX4_N_CS, *n* = 57; DDX4_N_CS–DDX4_N_, *n* = 74 (**d**). CTC distance is normalized by condensate radius (Methods). The ‘IDR1 (24 mer)–IDR2 (60 mer)’ represents respective fusion of the two IDRs to the cores. For example, FUS_N_–DDX4_N_ indicates the FUS_N_ is fused to 24-mer ferritin core and DDX4_N_ is fused to 60-mer I3-01^K129A^ core. All measurements are independent. Source data

**Extended Data Fig. 4:**
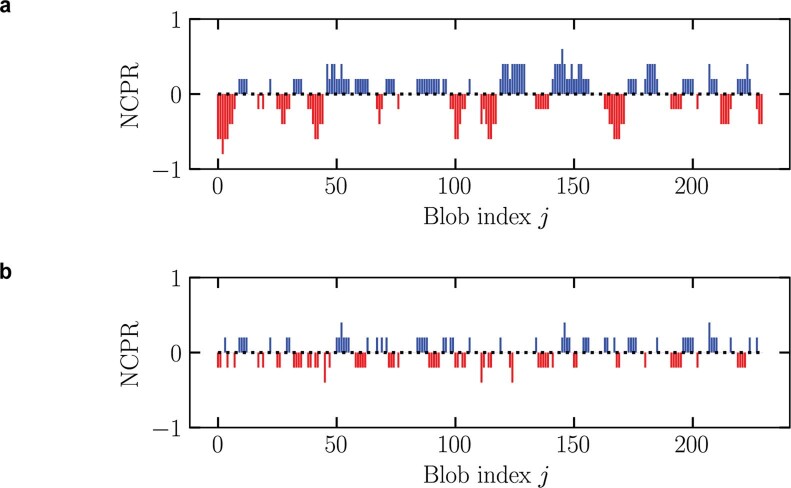
Comparison of the net charge per residue (NCPR) between DDX4_N_ and DDX4_N_CS. **a**,**b**. Net charge per residue for DDX4_N_ (**a**) and DDX4_N_CS (**b**). Source data

Our finding that the relative oligomerization of hnRNPA1_C_ is more important than that of FUS_N_ to the immiscibility of this system implies some asymmetry in how oligomerization can promote IDR immiscibility. We find a similar asymmetry in condensate pairs formed by FUS_N_ and DDX4_N_. In particular, we observe clear changes in the CTC distance between the two distinct phases, when one of the two IDRs (FUS_N_ or DDX4_N_) is fused to the 60-mer I3-01^K129A^ and the other to the 24-mer ferritin (Fig. 5a,b). To further examine the role of the precise IDR sequence in this oligomerization-driven asymmetric immiscibility, we tested a charge-scrambled version of DDX4_N_ (DDX4_N_CS (ref. ^13^; Extended Data Fig. 4) paired with FUS_N_ or DDX4_N_. One of the most striking examples of this asymmetry is observed with the FUS_N_/DDX4_N_CS pair: when FUS_N_ is highly oligomerized, two distinct phases are observed, while when DDX4_N_CS is more highly oligomerized, no distinct phases are observed. Indeed, condensate phases can be either fully miscible or immiscible, depending on the relative oligomerization state of the IDRs (Fig. 5a,c,d). This indicates that whether two different IDR sequences can drive two distinct and immiscible condensates depends both on specific sequence features and on their relative oligomerization states.

### Asymmetric multiphases from different oligomerization states

**Fig. 6:**
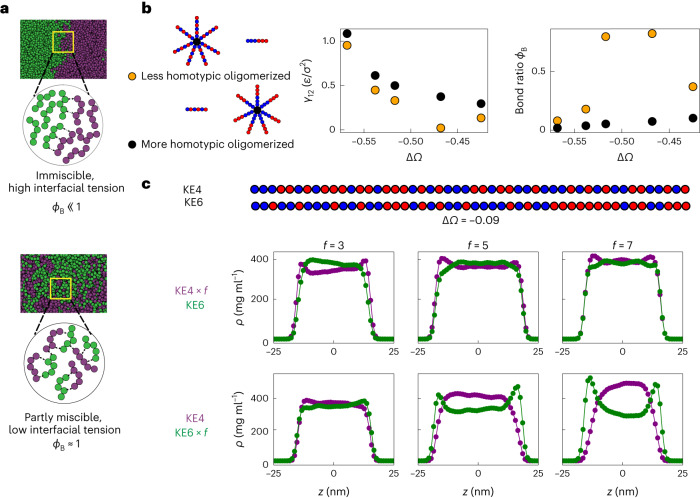
Asymmetric condensate immiscibility can arise from differential oligomerization. **a**, Snapshots of strongly and weakly immiscible two-phase systems demonstrate the relative variation in their interfacial tension. Schematics show how fewer heterotypic bonds are formed for the strongly immiscible system. **b**, Schematic illustrates the two configurations considered to study the effect of differential oligomerization on condensate miscibility. The interfacial tension γ_12_ in dimensionless units of *ε*/*σ*^2^, used as a measure of immiscibility, is consistently higher when the more homotypic IDR (more blocky charge patterning) in the pair is oligomerized. The *ε* represents the energy scale of interactions, while *σ* represents the size of a monomer bead. The bond ratio, defined as the ratio of unlike to like non-covalent bonds formed by the oligomerized IDR, is always lower for the system with the more homotypic IDR oligomerized. **c**, The binary mixture of KE4 and KE6 demonstrates how oligomerization asymmetrically tunes multiphase formation. Source data

We noticed that when the miscibility of DDX4_N_/DDX4_N_CS was assayed, a greater degree of immiscibility was observed when DDX4_N_ was more highly oligomerized (a median normalized CTC value of 0.18 versus 0.06; Fig. 5a,d). Since DDX4_N_ is well-established to possess stronger homotypic interactions than DDX4_N_CS (refs. ^13,15^), this suggests that the entropic ‘knob’ of oligomerization is coupled to the driving force provided by sequence interactions. Consistent with this hypothesis and our previous results (Fig. 4c), recent work using purified proteins suggests that hnRNPA1_C_ might have stronger homotypic interactions than FUS_N_ (refs. ^49,51^). To further examine this physical picture, we returned to our star polyampholyte simulation platform to elucidate how immiscibility depends on the identity of the oligomerized sequence (Fig. 6a). We ran two sets of simulations for each polyampholyte pair, where we studied the effect of oligomerizing each polyampholyte sequence on the miscibility behaviour. In the KE polyampholyte model, the strength of the homotypic interaction, as quantified by the critical temperature, has an approximately linear scaling with the SCD of the polyampholyte^37^. Thus, polyampholyte sequences with more blocky charge patterns have stronger homotypic interactions. Consistent with our hypothesis, we find that the degree of immiscibility is always higher when the polyampholyte with stronger homotypic interactions (that is, more blocky charge sequence) was oligomerized (Fig. 6b). For this set of simulations, we use the interfacial tension between the two polyampholyte phases as a proxy for the degree of immiscibility, which allows us to compare the relative change in miscibility when each IDR in the binary pair is oligomerized. To estimate the strength of the homotypic to heterotypic interaction, we used the ratio of heterotypic to homotypic bonds *ϕ*_B_ formed by the star polyampholytes as a proxy. In confirmation with our interfacial tension calculations, we find that the bond ratio (*ϕ*_B_) was consistently higher when the more homotypic IDR in the pair was oligomerized. These results suggest that oligomerization enhances the effective strength of homotypic interactions and penalizes the formation of heterotypic bonds. This energetic cost is substantially higher when the oligomerized polyampholyte has strong homotypic interactions, thereby leading to an asymmetric effect.

Based on our findings, we reasoned that even polyampholytes with small charge patterning differences should be asymmetrically immiscible at sufficiently high valences. To test this hypothesis, we simulated the binary pair KE4 and KE6, which have highly similar charge patterns (−Δ*Ω* = 0.09). We find that when KE4, which has weaker homotypic interactions, is oligomerized, the two components are perfectly miscible (Fig. 6c, top row). By contrast, when KE6 is oligomerized, the two component phases begin to demix, especially at higher valences (Fig. 6c, bottom row). Thus, while oligomerization can serve to amplify small sequence-dependent differences in interaction preference that underlie phase immiscibility, the impact of this effect depends on which sequence is oligomerized.

## Discussion

The molecular mechanisms behind the formation of biomolecular condensates and their resultant morphology and material properties have been the subject of intense study over the past decade. Most of this work has been directed towards understanding how biomolecular components in the cellular milieu can condense into single liquid-like phases. Yet, some of the most well-established endogenous condensates (for example, a nucleolus) are organized into multiple immiscible coexisting phases. Recent work has established that the organization of these coexisting immiscible phases is controlled by differences in interfacial tension, a mesoscale property, between the different phases^30^. However, the molecular mechanism underlying such mesoscale organization remains poorly understood, especially in vivo. In this study, we sought to investigate the role of oligomerization as a potential general molecular mechanism for driving the formation of multiphasic condensates.

To probe the biomolecular driving forces behind multiphase formation, we engineered orthogonal scaffolding ‘cores’ of distinct valences (24 mer and 60 mer) that enable the formation of synthetic condensates in vivo. The different valence states of the cores allowed us to probe the influence of oligomerization on the miscibility between distinct condensates. By fusing different archetypal IDRs to the cores, we found that the propensity to form multiple immiscible phases depends on the balance of sequence-encoded interactions. These findings suggest that biomolecular condensate components feature evolutionarily tuned sequence determinants that localize proteins to specific subcompartments.

A key finding, clearly seen in both our experimental and computational investigations, is that multiphasic structure depends asymmetrically on the oligomerization state of IDRs. For a given IDR pair, a greater degree of immiscibility should occur in configurations in which the IDR with the stronger homotypic interactions is fused to the higher valence core. Our results predict that the homotypic strengths of different IDRs within the intracellular context are ordered as follows: hnRNPA1_C_ > FUS_N_ > DDX4_N_. Interestingly, we found that in living cells, DDX4_N_ has the weakest homotypic interactions among the tested IDRs, which constrasts with in vitro measurements^13,15^. Our simulations also revealed that oligomerization increases the propensity to form multiphase structures by amplifying the relative difference in the strength of homotypic versus heterotypic interactions. This leads to an emergent asymmetry, with a stronger driving force for multiphase organization when more homotypic IDRs are oligomerized. Thus, both IDR sequence and relative degree of oligomerization contribute to the complex interplay of mixing entropy and molecular interactions that underlies multiphase condensate organization.

How might the cell have naturally exploited the effects of such differential oligomerization states to evolve biological functions? There is growing evidence that proteins implicated in phase separation feature structured domains that enable binding to RNA or DNA^52,53^. Indeed, RNA binding proteins feature prominently in multiphasic condensates like the nucleolus (NPM1)^20,21,30,54^ and stress granule/P-body (G3BP1/2)^22,55,56^. Truncation mutants of NPM1 lacking either its C-terminal oligomerization domain or N-terminal RNA recognition motif were found to mislocalize when expressed in cells^57^. Similarly, G3BP1 mutants lacking an RNA binding domain (RBD) were unable to form stress granules when expressed in double knockout lines^22^. Furthermore, transcriptional inhibition is well known to result in aberrant nucleolar morphologies and the formation of nucleolar caps^31^. Indeed, it has been suggested that RNA could potentially act as a biological super-scaffold that can tunably drive multiphase formation^58^. Since RBDs can be regulated through post-translational modifications, we speculate that living cells could dynamically modulate the oligomerization state of recruited RBDs to drive multiphase condensate formation. Intriguingly, our results also suggest oligomerization as a potential mechanism for altering the spatial localization of condensate components. We speculate that, in the nucleolus, this could potentially be a tunable biophysical mechanism by which fully processed ribosomes are efficiently fluxed out into the nucleoplasm.

The ability to control the miscibility between condensates through the interplay of sequence identity and oligomerization could be exploited for the design of synthetic organelles. Orthogonal scaffolds, potentially actuated externally with light, with chemicals or through other means, could enable the inducible formation of multiphasic condensates for the spatial organization of enzymatic reactions. One possible future strategy, for example, is to explore optogenetic methods that temporally switch miscible components of a single homogeneous condensate into demixed components of a spatially segregated, multiphase structure. Such potential engineering applications represent an exciting new frontier, advanced through these and future discoveries, of the increasingly rich ways in which living cells harness oligomerization to spatially organize condensates and control their associated biological functions.

## Methods

### Cell culture and cell-line generation

The U2OS (a kind gift from the Mark Groudine lab, Fred Hutchinson Cancer Research Center) and Lenti-X 293T (Takara) cells were cultured in a growth medium consisting of Dulbecco’s modified Eagle’s medium (Gibco), 10% foetal bovine serum (Atlanta Biologicals) and 10 U ml^–1^ penicillin–streptomycin (Gibco) and incubated at 37 °C and 5% CO_2_ in a humidified incubator.

### Lentiviral transduction

For Corelet and NPM1 overexpression, lentiviruses containing desired constructs were produced by transfecting the plasmid along with helper plasmids VSVG and PSP (a kind gift from the Marc Diamond lab, UT Southwestern) into HEK293T cells with Lipofectamine 3000 (Invitrogen). Virus was collected 2–3 days after transfection and used to infect wild-type U2OS. Lentivirus transduction was performed in 96-well plates. Three days following lentivirus application to cells at low confluency, cells were passaged for stable maintenance or directly to 96-well fibronectin-coated glass bottom dishes for live cell microscopy. The infected cells were imaged no earlier than 72 h after infection.

### Yeast plasmid construction

All integration and 2μ plasmids were constructed based on the pJLA vectors using either the restriction digest and ligation method with T4 DNA ligase (NEB) or the In-Fusion HD cloning kit (Takara Bio). The following restriction enzymes were used for cloning: MreI (Thermo Fisher Scientific), SpeI-HF (NEB), BamHI-HF (NEB), NotI-HF (NEB), AgeI-HF (NEB), AscI (NEB), XhoI (NEB) and SacI-HF (NEB). Promoters (CCW12p, HHF2p and RPL18Bp) and terminators (TDH1t, ENO2t and ENO1t) that are not in the pJLA vectors were obtained from a published yeast toolkit on Addgene^50^. Cloned plasmids were transformed into *E. coli* Stellar Competent Cells (Takara Bio), from which single colonies were isolated from Luria–Bertani agar plates supplemented with ampicillin. Next, colonies carrying correct clones were identified by colony polymerase chain reaction using OneTaq Hot Start Quick-Load 2X Master Mix (NEB), and plasmids were purified from overnight culture in Luria–Bertani containing 150 μg ml^–1^ ampicillin at 37 °C. All cloned plasmids were verified by Sanger sequencing (Genewiz). Recombinant DNA (I3-01, O3-33 and DDX4_N_CS) were codon-optimized for *S. cerevisiae* using the IDT codon optimization tool and purchased as gBlocks gene fragments from IDT.

### Yeast transformation and culture

Integration plasmids were linearized with PmeI (NEB) and transformed into yeast using the standard lithium acetate method^59^. *S. cerevisiae* CEN.PK2-1C (MATa, ura3-52, trp1-289, leu2-3112, his3Δ1, MAL2-8c, SUC2) was used as the background to construct all yeast strains used in this study. Transformants were selected on synthetic complete (SC) dropout agar plates lacking appropriate amino acid for auxotrophic marker selection, supplemented with 2% w/v glucose. To verify the integration of the linearized DNA construct into the yeast genome, single colonies were isolated and grown in 1 ml SC media lacking appropriate amino acid with 2% w/v glucose overnight at 30 °C in 24-well plates covered with aluminium foil. The next day, the overnight culture was diluted 1:50 into fresh media in 24-well plates and grown at 30 °C for 4 h until the early exponential phase for imaging (the optical density of a sample measured at a wavelength of 600 nm (OD_600_) was between 1 and 2). Yeast strains were stored as 20% v/v glycerol stocks at −80 °C.

### Airyscan microscopy

The super-resolution fluorescence images were taken with a ×100 α Plan-Apochromat 1.46 Oil DIC M27 objective on a Zeiss LSM 980 Airyscan 2.0 microscope using the Airyscan SR mode. Imaging was performed using Zeiss Zen Blue v.3.2 software. To image a multi-channel *z*-stack image, frame switching was used, and the entire *z* stack was imaged per track before switching the channel. The 405 nm, 488 nm, 561 nm and 639 nm lasers were used to image mTagBFP2, GFP, mCherry and MemBrite 640/660 fix dye (Biotium) in cells, respectively. The point spread function was verified using TetraSpeck microspheres (Thermo Fisher T7279).

### Quantitative microscopy to determine the valence of NPM1-Corelets

Quantitative microscopy was performed using a Zeiss LSM 980 confocal microscope equipped with a spectral array detector (32-element cooled GaAsP array functioning as a spectral confocal). The spectral array detector enabled fluorescence correlation spectroscopy for determining the concentration of fluorophore-tagged proteins (GFP and mCherry) in live cells. First, the effective confocal volume (*V*_eff_) of the objective (×60, 1.43 numerical aperture, oil, structural parameter = 7) was determined at a *z* height close to the common cell imaging plane. Following the established protocol^60^, the *V*_eff_ values when using 488 nm and 561 nm lasers were determined with aqueous solutions of Atto 488 (concentration = 200 nM) and Alexa Fluor 568 (concentration = 50 nM), respectively, at 37 °C.

In cells that stably express GFP–P2A–mCherry (where P2A is the picornavirus-derived self-cleaving 2A sequence), we used fluorescence correlation spectroscopy to determine the number of diffusing particles in a corresponding *V*_eff_. Using the *V*_eff_ measured using dye solutions, the concentrations of GFP and mCherry were determined. A series of images were then collected using the Zeiss LSM online fingerprinting with a wide range of laser power (0.01–5%) and gain (650–850 V). Using the image intensity at different acquisition settings, intensity–concentration calibration curves were determined (at least three different cells for each fluorophore). The concentrations of NPM1–mCherry–SspB and NLS–iLID–GFP–FTH1 were determined from these calibration curves, similar to previously reported methods^40^. All the multicolour mammalian cell images and time series were collected using the spectral unmixing of mTagBFP2, GFP and mCherry using the online fingerprinting module on the Zeiss LSM. The optogenetic activation was performed with lasers of 405 nm (intensity = 0.2%) and 488 nm (intensity = 0.3%).

### Yeast sample preparation for fixed-cell imaging

For fixed-cell imaging experiments on the Zeiss LSM 980 with Airyscan, strains were streaked from glycerol stocks, cultured as described above and imaged on the 96-well plate. The wells were coated with 50 μl solution of 1 mg ml^–1^ concanavalin A (ConA; Sigma-Aldrich L7647) in 20 mM NaOAc for yeast immobilization, as previously described^61^. The yeast culture was diluted to an OD_600_ of 1, and 100 μl of the diluted culture was added to each well. Yeast surface staining using the MemBrite 640/660 fix dye was done after immobilization and prior to fixation following the manufacturer’s protocol. Yeast was fixed with 100 μl 4% paraformaldehyde in phosphate-buffered saline (PBS) at room temperature for 20 min. Finally, each well was washed twice with PBS and 100 μl PBS was added before imaging.

### Normalized CTC distance calculation

The condensate sizes and distances in yeast imaging experiments were measured using the Distance Analysis (DiAna) plugin in Fiji ImageJ2 (refs. ^62,63^). The condensates in each channel were segmented using the iterative threshold method with a minimum size of 50 pixels and a minimum threshold of 20% of the average maximum intensity across images of the same well. Condensate sizes and the CTC distances between overlapping condensates were obtained using the DiAna colocalization analysis. Outputs of the DiAna colocalization analysis were individually verified. The CTC distance between overlapping condensates was then normalized by the sum of the radii of the two condensates. To account for noise in the measurements, the bin width was set to 0.1 in the frequency distribution.

### Simulation methods

**Extended Data Fig. 5:**
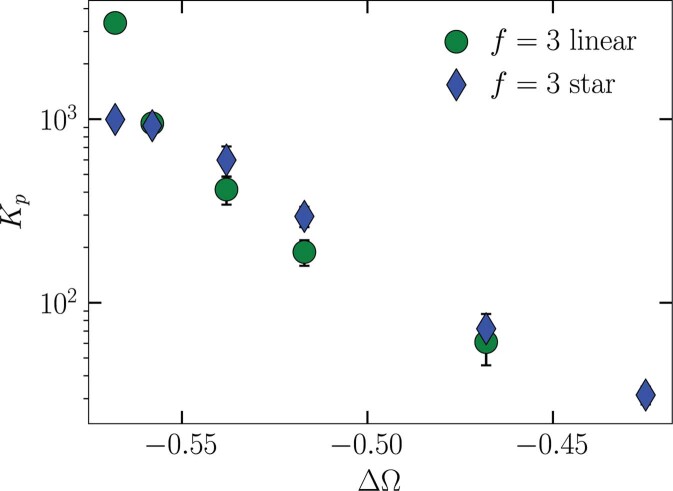
Variation of the partitioning as a function of ΔΩ for star and linear oligomerization of component *p*. For star oligomerization, polyampholytes were connected to a central hard-sphere bead. For linear oligomerization, polyampholytes were connected to each other end to end. Source data

Direct coexistence simulations were performed to estimate the relative miscibility of KE polyampholyte pairs^35,37^. To model molecular interactions between the monomers, we used the hydrophobicity scale-implicit solvent model^64^. Multivalency in polyampholytes was modelled in our simulations by covalently linking polyampholytes (using hydrophobicity scale bond parameters) to a central hard-sphere bead with diameter 1.2 Å, roughly double the diameter of a lysine or glutamate bead. The effect of star versus linear oligomerization is considered in Extended Data Fig. 5 and was found to have only a minor effect. All simulation runs were performed using HOOMD-blue^65^.

Initial configurations were generated by randomly placing both polyampholyte species, at equal mass fraction, in a 50 nm × 50 nm × 50 nm cubic box and performing a short run in which the simulation box was compressed to either 20 nm × 20 nm × 20 nm or 25 nm × 25 nm × 25 nm, depending on system size and the valence of the polyampholytes. The system sizes used for different valence states are described in Extended Data Table 3. Larger simulation boxes were used for oligomerized polyampholytes to accomodate increased system sizes (Extended Data Table 3). In accordance with the direct coexistence method^66,67^, the *z* dimension of the box was then expanded to 125 nm by adding empty space to either side, and an equilibration run was performed at constant particle number *N*, system volume *V* and temperature *T* (*NVT*). Finally, using the equilibrated *NVT* configurations, *NPAT* (keeping particle number *N*, pressure *P*, cross-sectional area of box *A* and temperature *T*) direct coexistence runs were performed to obtain the miscibility behaviour. A time step of 10 fs was used for all runs, and both the *NVT* and *NPAT* simulations were run for 5 µs. All simulations were run at a temperature of 250 K, which is lower than the critical temperature of sequence KE1 (the polyampholyte sequence with the lowest critical temperature). For *NVT* simulations, a Langevin thermostat with a friction coefficient of 1 ps^−1^ was used, while for *NPAT* simulations, a Martyna–Tobias–Klein barostat was used with coupling constants *τ* = 0.5 and *τ*_p_ = 0.5, representing the coupling constant for the thermostat and barostat, respectively.

To obtain coexistence data, density profiles for both polyampholyte species were recorded along the *z* dimension and averaged over the entire trajectory. For estimating the partitioning, we fit a hyperbolic tangent function to the density profile to estimate the concentration of the dense and dilute phases, as well as the spatial extent of each phase in the *z* dimension. Partitioning was then estimated as described previously. For comparing the miscibility behaviour of differentially oligomerized polyampholytes, there is no reference phase that can be consistently used to estimate the partitioning since both sequence and valence are varying. Thus, the interfacial tension between the two polyampholyte phases was used as a measure of the immiscibility. Interfacial tension (Fig. 6b) was calculated from the components of the pressure tensor as previously described in the literature^67,68^. All simulation snapshots were generated using the ‘freud’ Python library^69^.

### Reporting summary

Further information on research design is available in the Nature Portfolio Reporting Summary linked to this article.

## Online content

Any methods, additional references, Nature Portfolio reporting summaries, source data, extended data, supplementary information, acknowledgements, peer review information; details of author contributions and competing interests; and statements of data and code availability are available at 10.1038/s41557-024-01456-6.

## Supplementary information

Supplementary Information Supplementary Tables 1 and 2.

Reporting Summary

## Source data

Source Data Fig. 1 Statistical source data.

Source Data Fig. 2 Uncropped representative images.

Source Data Fig. 3 Statistical source data.

Source Data Fig. 4 Statistical source data.

Source Data Fig. 5 Statistical source data.

Source Data Fig. 6 Statistical source data.

Source Data Extended Data Fig. 1 Statistical source data.

Source Data Extended Data Fig. 2 Statistical source data.

Source Data Extended Data Fig. 3 Statistical source data.

Source Data Extended Data Fig. 4 Statistical source data.

Source Data Extended Data Fig. 5 Statistical source data.

## Figures and Tables

**Extended Data Table 1:**
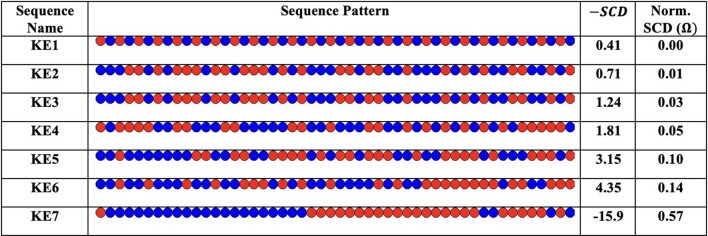
Polyampholyte sequences studied

Red beads represent negatively charged glutamate (E) residues while blue beads represent positively charged lysine residues (K). We note that KE1, KE6 and KE7 are identical SV1, SV15 and SV28 from the previously established SV system of model polyampholyte sequences^35^.

**Extended Data Table 2:**
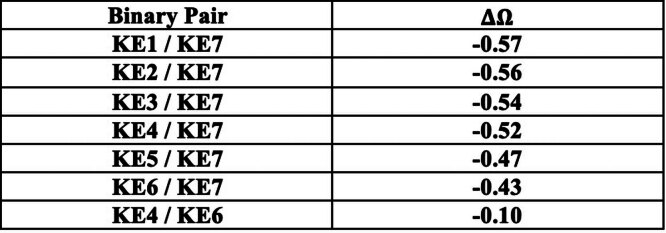
Polyampholyte sequences binary pairs studied All polyampholyte pairs investigated in this work alongside the differences in their normalized SCD values (ΔΩ).

**Extended Data Table 3:**
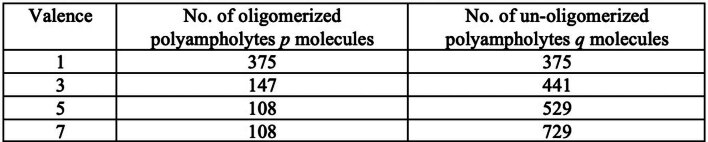
System sizes for simulations Number of oligomerized and unoligomerized polyampholyte molecules present in simulation box at each valence investigated.

## Data Availability

The data that support the findings of this study are included in this Article and in the Supplementary Information. Source data are provided with this paper.

## Code availability

Codes for setting up and performing the molecular dynamics simulations associated with this work are publicly available via GitHub at https://github.com/SoftLivingMatter/Asymmetric_Immiscibility_Simulations/.
