# Supplementary material for: Asymmetric oligomerization state and sequence patterning can tune multiphase condensate miscibility
Source: Nat Chem. 2024 Feb 21;16(7):1073–82. doi: 10.1038/s41557-024-01456-6 (PMC11230906; doi:10.1038/s41557-024-01456-6)
Supplement: Supplementary file 1 — Supplementary Tables 1 and 2. [file 41557_2024_1456_MOESM1_ESM.pdf]

nature chemistry

Article

https://doi.org/10.1038/s41557-024-01456-6

Asymmetric oligomerization state and sequence patterning can tune multiphase condensate miscibility

In the format provided by the authors and unedited

Table of Contents

***Supplementary Table 1. Yeast strains used in this study.* 1**

***Supplementary Table 2. Plasmids used in this study*.. 4**

***Supplementary References* 5**

Supplementary Table 1. Yeast strains used in this study.

Strain	Description	Genotype	Source
CEN.PK2-1C	Wild-type *Saccharomyces cerevisiae*	*MATa ura3-52 trp1-289 leu2-3,112 his3-1 MAL2-8c SUC2*	[1]
yKX371	Investigate core orthogonality	*his3::HIS3*-TEF1p_FUS$_N$-mCherry-FTH1_TDH1t<br>*leu2::LEU2*-PGK1p_GFP-FTH1_CYC1t	This study
yKX372	Investigate core orthogonality	*his3::HIS3*-TEF1p_FUS$_N$-mCherry-FTH1_TDH1t<br>*leu2::LEU2*-PGK1p_GFP-I301(K129A)_ENO2t	This study
yKX373	Investigate core orthogonality	*his3::HIS3*-TEF1p_FUS$_N$-mCherry-FTH1_TDH1t<br>*leu2::LEU2*-PGK1p_O333-GFP_ENO2t	This study
yKX374	Investigate core orthogonality	*his3::HIS3*-TEF1p_FUS$_N$-mCherry-I301(K129A)_TDH1t<br>*leu2::LEU2*-PGK1p_GFP-FTH1_CYC1t	This study
yKX375	Investigate core orthogonality	*his3::HIS3*-TEF1p_FUS$_N$-mCherry-I301(K129A)_TDH1t<br>*leu2::LEU2*-PGK1p_GFP-I301(K129A)_ENO2t	This study
yKX376	Investigate core orthogonality	*his3::HIS3*-TEF1p_FUS$_N$-mCherry-I301(K129A)_TDH1t<br>*leu2::LEU2*-PGK1p_O333-GFP_ENO2t	This study
yKX377	Investigate core orthogonality	*his3::HIS3*-TEF1p_O333-mCherry-hnRNPA1$_C$_TDH1t<br>*leu2::LEU2*-PGK1p_GFP-FTH1_CYC1t	This study
yKX378	Investigate core orthogonality	*his3::HIS3*-TEF1p_O333-mCherry-hnRNPA1$_C$_TDH1t<br>*leu2::LEU2*-PGK1p_GFP-I301(K129A)_ENO2t	This study
yKX379	Investigate core orthogonality	*his3::HIS3*-TEF1p_O333-mCherry-hnRNPA1$_C$_TDH1t<br>*leu2::LEU2*-PGK1p_O333-GFP_ENO2t	This study
yKX172	Synthetic IDR-core proteins immiscibility	*his3::HIS3*-TEF1p_FUS$_N$-mCherry-I301(K129A)_TDH1t<br>*leu2::LEU2*-PGK1p_FUS$_N$-GFP-FTH1_ENO2t	This study
yKX173	Synthetic IDR-core proteins immiscibility	*his3::HIS3*-TEF1p_DDX4$_N$-mCherry-I301(K129A)_TDH1t<br>*leu2::LEU2*-PGK1p_FUS$_N$-GFP-FTH1_ENO2t	This study
yKX294	Synthetic IDR-core proteins immiscibility	*his3::HIS3*-TEF1p_DDX4$_N$CS-mCherry-I301(K129A)_TDH1t<br>*leu2::LEU2*-PGK1p_FUS$_N$-GFP-FTH1_ENO2t	This study
yKX175	Synthetic IDR-core proteins immiscibility	*his3::HIS3*-TEF1p_FUS$_N$-mCherry-I301(K129A)_TDH1t<br>*leu2::LEU2*-PGK1p_DDX4$_N$-GFP-FTH1_ENO2t	This study
yKX176	Synthetic IDR-core proteins immiscibility	*his3::HIS3*-TEF1p_DDX4$_N$-mCherry-I301(K129A)_TDH1t<br>*leu2::LEU2*-PGK1p_DDX4$_N$-GFP-FTH1_ENO2t	This study
yKX297	Synthetic IDR-core proteins immiscibility	*his3::HIS3*-TEF1p_DDX4$_N$CS-mCherry-I301(K129A)_TDH1t<br>*leu2::LEU2*-PGK1p_DDX4$_N$-GFP-FTH1_ENO2t	This study
yKX296	Synthetic IDR-core proteins immiscibility	*his3::HIS3*-TEF1p_FUS$_N$-mCherry-I301(K129A)_TDH1t<br>*leu2::LEU2*-PGK1p_DDX4$_N$CS-GFP-FTH1_ENO2t	This study
yKX331	Synthetic IDR-core proteins immiscibility	*his3::HIS3*-TEF1p_DDX4$_N$CS-mCherry-I301(K129A)_TDH1t<br>*leu2::LEU2*-PGK1p_DDX4$_N$CS-GFP-FTH1_ENO2t	This study
yKX332	Synthetic IDR-core proteins immiscibility	*his3::HIS3*-TEF1p_DDX4$_N$-mCherry-I301(K129A)_TDH1t<br>*leu2::LEU2*-PGK1p_DDX4$_N$CS-GFP-FTH1_ENO2t	This study

Strain	Description	Genotype	Source
yKX335	Oligomerization-driven immiscibility	*his3::HIS3*-TEF1p_O333-mCherry-hnRNPA1$_{C}$_TDH1t<br>*leu2::LEU2*-PGK1p_FUS$_{N}$-GFP-FTH1_ENO2t_<br>Rev(TEF1p_mTagBFP2-FTH1_ACT1t)	This study
yKX336	Oligomerization-driven immiscibility	*his3::HIS3*-TEF1p_O333-mCherry-hnRNPA1$_{C}$_TDH1t<br>*leu2::LEU2*-PGK1p_FUS$_{N}$-GFP-FTH1_ENO2t_<br>Rev(TEF1p_mTagBFP2-FTH1_ACT1t)<br>*trp1::TRP1*-CCW12p_mTagBFP2-FTH1_ENO1t	This study
yKX337	Oligomerization-driven immiscibility	*his3::HIS3*-TEF1p_O333-mCherry-hnRNPA1$_{C}$_TDH1t<br>*leu2::LEU2*-PGK1p_FUS$_{N}$-GFP-FTH1_ENO2t_<br>Rev(TEF1p_mTagBFP2-FTH1_ACT1t)<br>*trp1::TRP1*-CCW12p_mTagBFP2-FTH1_ENO1t_<br>Rev(TEF1p_mTagBFP2-FTH1_ACT1t)	This study
yKX349	Oligomerization-driven immiscibility	*his3::HIS3*-TEF1p_O333-mCherry-hnRNPA1$_{C}$_TDH1t_<br>Rev(TDH3p_O333-mCherry_ENO1t)<br>*leu2::LEU2*-PGK1p_FUS$_{N}$-GFP-FTH1_ENO2t_<br>Rev(TEF1p_mTagBFP2-FTH1_ACT1t)	This study
yKX355	Oligomerization-driven immiscibility	*his3::HIS3*-TEF1p_O333-mCherry-hnRNPA1$_{C}$_TDH1t_<br>Rev(TDH3p_O333-mCherry_ENO1t)<br>*leu2::LEU2*-PGK1p_FUS$_{N}$-GFP-FTH1_ENO2t_<br>Rev(TEF1p_mTagBFP2-FTH1_ACT1t)<br>*trp1::TRP1*-CCW12p_mTagBFP2-FTH1_ENO1t	This study
yKX356	Oligomerization-driven immiscibility	*his3::HIS3*-TEF1p_O333-mCherry-hnRNPA1$_{C}$_TDH1t_<br>Rev(TDH3p_O333-mCherry_ENO1t)<br>*leu2::LEU2*-PGK1p_FUS$_{N}$-GFP-FTH1_ENO2t_<br>Rev(TEF1p_mTagBFP2-FTH1_ACT1t)<br>*trp1::TRP1*-CCW12p_mTagBFP2-FTH1_ENO1t_<br>Rev(TEF1p_mTagBFP2-FTH1_ACT1t)	This study
yKX358	Oligomerization-driven immiscibility	*his3::HIS3*-TEF1p_O333-mCherry-hnRNPA1$_{C}$_TDH1t_<br>Rev(HHF2p_O333-mCherry_ENO1t)<br>*leu2::LEU2*-PGK1p_FUS$_{N}$-GFP-FTH1_ENO2t_<br>Rev(TEF1p_mTagBFP2-FTH1_ACT1t)	This study
yKX359	Oligomerization-driven immiscibility	*his3::HIS3*-TEF1p_O333-mCherry-hnRNPA1$_{C}$_TDH1t_<br>Rev(HHF2p_O333-mCherry_ENO1t)<br>*leu2::LEU2*-PGK1p_FUS$_{N}$-GFP-FTH1_ENO2t_<br>Rev(TEF1p_mTagBFP2-FTH1_ACT1t)<br>*trp1::TRP1*-CCW12p_mTagBFP2-FTH1_ENO1t	This study
yKX360	Oligomerization-driven immiscibility	*his3::HIS3*-TEF1p_O333-mCherry-hnRNPA1$_{C}$_TDH1t_<br>Rev(HHF2p_O333-mCherry_ENO1t)<br>*leu2::LEU2*-PGK1p_FUS$_{N}$-GFP-FTH1_ENO2t_<br>Rev(TEF1p_mTagBFP2-FTH1_ACT1t)<br>*trp1::TRP1*-CCW12p_mTagBFP2-FTH1_ENO1t_<br>Rev(TEF1p_mTagBFP2-FTH1_ACT1t)	This study
yKX362	Oligomerization-driven immiscibility	*his3::HIS3*-TEF1p_O333-mCherry-hnRNPA1c_TDH1t_<br>Rev(RPL18Bp_O333-mCherry_ENO1t)<br>*leu2::LEU2*-PGK1p_FUSn-GFP-FTH1_ENO2t_<br>Rev(TEF1p_mTagBFP2-FTH1_ACT1t)	This study

Strain	Description	Genotype	Source
yKX363	Oligomerization-driven immiscibility	*his3::HIS3*-TEF1p_O333-mCherry-hnRNPA1c_TDH1t_ Rev(RPL18Bp_O333-mCherry_ENO1t)<br>*leu2::LEU2*-PGK1p_FUSn-GFP-FTH1_ENO2t_ Rev(TEF1p_mTagBFP2-FTH1_ACT1t)<br>*trp1::TRP1*-CCW12p_mTagBFP2-FTH1_ENO1t	This study
yKX364	Oligomerization-driven immiscibility	*his3::HIS3*-TEF1p_O333-mCherry-hnRNPA1c_TDH1t_ Rev(RPL18Bp_O333-mCherry_ENO1t)<br>*leu2::LEU2*-PGK1p_FUSn-GFP-FTH1_ENO2t_ Rev(TEF1p_mTagBFP2-FTH1_ACT1t)<br>*trp1::TRP1*-CCW12p_mTagBFP2-FTH1_ENO1t_ Rev(TEF1p_mTagBFP2-FTH1_ACT1t)	This study

Supplementary Table 2. Plasmids used in this study.

Plasmid	Description	Source
**Yeast**		
pKX020	Amp^R, HIS3 integration, TEF1p_DDX4$_N$CS-mCherry-I301(K129A)_TDH1t	This study
pKX100	Amp^R, HIS3 integration, TEF1p_FUSn-mCherry-FTH1_TDH1t	This study
pKX101	Amp^R, LEU2 integration, PGK1p_GFP-FTH1_CYC1t	This study
pKX117	Amp^R, HIS3 integration, TEF1p_FUS$_N$-mCherry-I301(K129A)_TDH1t	This study
pKX119	Amp^R, HIS3 integration, TEF1p_DDX4$_N$-mCherry-I301(K129A)_TDH1t	This study
pKX121	Amp^R, LEU2 integration, PGK1p_FUS$_N$-GFP-FTH1_ENO2t	This study
pKX123	Amp^R, LEU2 integration, PGK1p_DDX4$_N$-GFP-FTH1_ENO2t	This study
pKX180	Amp^R, LEU2 integration, PGK1p_GFP-I301(K129A)_ENO2t	This study
pKX328	Amp^R, HIS3 integration, TEF1p_O333-mCherry-hnRNPA1$_C$_TDH1t	This study
pKX338	Amp^R, LEU2 integration, PGK1p_DDX4$_N$CS-GFP-FTH1_ENO2t	This study
pKX393	Amp^R, 2μ, URA3, TEF1p_mTagBFP2-FTH1_ACT1t	This study
pKX394	Amp^R, LEU2 integration, PGK1p_FUS$_N$-GFP-FTH1_ENO2t_ Rev(TEF1p_mTagBFP2-FTH1_ACT1t)	This study
pKX395	Amp^R, TRP1 integration, CCW12p_mTagBFP2-FTH1_ENO1t	This study
pKX396	Amp^R, TRP1 integration, CCW12p_mTagBFP2-FTH1_ENO1t_ Rev(TEF1p_mTagBFP2-FTH1_ACT1t)	This study
pKX399	Amp^R, HIS3 integration, TDH3p_O333-mCherry_ENO1t	This study
pKX400	Amp^R, HIS3 integration, TEF1p_O333-mCherry-hnRNPA1$_C$_TDH1t_ Rev(TDH3p_O333-mCherry_ENO1t)	This study
pKX430	Amp^R, HIS3 integration, HHF2p_O333-mCherry_ENO1t	This study
pKX431	Amp^R, HIS3 integration, RPL18Bp_O333-mCherry_ENO1t	This study
pKX433	Amp^R, HIS3 integration, TEF1p_O333-mCherry-hnRNPA1$_C$_TDH1t_ Rev(HHF2p_O333-mCherry_ENO1t)	This study
pKX434	Amp^R, HIS3 integration, TEF1p_O333-mCherry-hnRNPA1$_C$_TDH1t_ Rev(RPL18Bp_O333-mCherry_ENO1t)	This study
pKX452	Amp^R, LEU2 integration, PGK1p_O333-GFP_ENO2t	This study
**Mammalian**		
	FM5::NPM1-mCherry-sspB	This study
	FM5::NLS-iLID-GFP-FTH1	This study
	FM5::mTagBFP2-NPM1	This study

Supplementary References

1. Entian, K.-D. & Kötter, P. 25 Yeast Genetic Strain and Plasmid Collections. in *Methods in Microbiology* (eds. Stansfield, I. & Stark, M., Jr) vol. 36 629–666 (Academic Press, 2007).
