# Supplementary material for: Asymmetric oligomerization state and sequence patterning can tune multiphase condensate miscibility
Source: Nat Chem. 2024 Feb 21;16(7):1073–82. doi: 10.1038/s41557-024-01456-6 (PMC11230906; doi:10.1038/s41557-024-01456-6)
Supplement: Supplementary file 2 — Reporting Summary [file 41557_2024_1456_MOESM2_ESM.pdf]

nature portfolio

Corresponding author(s): Clifford P. Brangwynne

Last updated by author(s): Nov 16, 2023

Reporting Summary

Nature Portfolio wishes to improve the reproducibility of the work that we publish. This form provides structure for consistency and transparency in reporting. For further information on Nature Portfolio policies, see our Editorial Policies and the Editorial Policy Checklist.

Statistics

For all statistical analyses, confirm that the following items are present in the figure legend, table legend, main text, or Methods section.

n/a	Confirmed	
☐	☒	The exact sample size (n) for each experimental group/condition, given as a discrete number and unit of measurement
☐	☒	A statement on whether measurements were taken from distinct samples or whether the same sample was measured repeatedly
☒	☐	The statistical test(s) used AND whether they are one- or two-sided<br>*Only common tests should be described solely by name; describe more complex techniques in the Methods section.*
☒	☐	A description of all covariates tested
☒	☐	A description of any assumptions or corrections, such as tests of normality and adjustment for multiple comparisons
☐	☒	A full description of the statistical parameters including central tendency (e.g. means) or other basic estimates (e.g. regression coefficient) AND variation (e.g. standard deviation) or associated estimates of uncertainty (e.g. confidence intervals)
☒	☐	For null hypothesis testing, the test statistic (e.g. F, t, r) with confidence intervals, effect sizes, degrees of freedom and P value noted<br>*Give P values as exact values whenever suitable.*
☒	☐	For Bayesian analysis, information on the choice of priors and Markov chain Monte Carlo settings
☒	☐	For hierarchical and complex designs, identification of the appropriate level for tests and full reporting of outcomes
☐	☒	Estimates of effect sizes (e.g. Cohen's d, Pearson's r), indicating how they were calculated

Our web collection on statistics for biologists contains articles on many of the points above.

Software and code

Policy information about availability of computer code

Data collection	Zeiss Zen Blue 3.2 software, HOOMD-blue 2.9.7,
Data analysis	Fiji (ImageJ 1.52p), Graphpad Prism, and Python were used for data analysis as detailed in the manuscript.

For manuscripts utilizing custom algorithms or software that are central to the research but not yet described in published literature, software must be made available to editors and reviewers. We strongly encourage code deposition in a community repository (e.g. GitHub). See the Nature Portfolio guidelines for submitting code & software for further information.

Data

Policy information about availability of data

All manuscripts must include a data availability statement. This statement should provide the following information, where applicable:

- Accession codes, unique identifiers, or web links for publicly available datasets
- A description of any restrictions on data availability
- For clinical datasets or third party data, please ensure that the statement adheres to our policy

The data that support the findings of this study are available included in this Article, in the source data files and the Supplementary Information.

Human research participants

Policy information about studies involving human research participants and Sex and Gender in Research.

Reporting on sex and gender	Not Applicable
Population characteristics	Not Applicable
Recruitment	Not Applicable
Ethics oversight	Not Applicable

Note that full information on the approval of the study protocol must also be provided in the manuscript.

Field-specific reporting

Please select the one below that is the best fit for your research. If you are not sure, read the appropriate sections before making your selection.

☒ Life sciences ☐ Behavioural & social sciences ☐ Ecological, evolutionary & environmental sciences

For a reference copy of the document with all sections, see nature.com/documents/nr-reporting-summary-flat.pdf

Life sciences study design

All studies must disclose on these points even when the disclosure is negative.

Sample size	Sample size was not predetermined; for the distribution study, we analyzed collected data in real-time during experiment until we not longer observed significant changes in the data distribution. In general for microscopy experiment, we tried to captured multiple field-of-view in each well of each biological replicate.
Data exclusions	No data excluded
Replication	Data are collected in multiple locations within the well to ensure accurate capture of phenomenon of interest. To ensure reproducibility, we randomly selected yeast strains to image on different days. All attempts at replicating the population distribution were successful.
Randomization	This is not relevant in our study. In our experiments, we didn't need to allocate different yeast strains/cell lines into group. It is actually very important for each yeast strain/cell line to remain distinct from one another.
Blinding	This is not relevant in our study. In our experiment, we named our yeast strains and cell lines with unique name/indentifier, and we reported data collected in individual yeast strains and cell lines.

Reporting for specific materials, systems and methods

We require information from authors about some types of materials, experimental systems and methods used in many studies. Here, indicate whether each material, system or method listed is relevant to your study. If you are not sure if a list item applies to your research, read the appropriate section before selecting a response.

Materials & experimental systems

n/a	Involved in the study	
☒	☐	Antibodies
☐	☒	Eukaryotic cell lines
☒	☐	Palaeontology and archaeology
☒	☐	Animals and other organisms
☒	☐	Clinical data
☒	☐	Dual use research of concern

Methods

n/a	Involved in the study	
☒	☐	ChIP-seq
☒	☐	Flow cytometry
☒	☐	MRI-based neuroimaging

Eukaryotic cell lines

Policy information about cell lines and Sex and Gender in Research

Cell line source(s)	U2OS (Mark Groudine lab, Fred Hutchinson Cancer Research Center), HEK293T (LentiX from Takara Bio), S. cerevisiae:

Cell line source(s)	CEN.PK-1C (Euroscarf#30000A)
Authentication	Cell lines were not authenticated. Visual confirmation was done for U2OS, HEK293T and yeast cell lines
Mycoplasma contamination	Cell lines were not tested for mycoplasma contamination. No contamination was visually observed
Commonly misidentified lines<br>(See ICLAC register)	No commonly misidentified cell lines were used
