# Supplementary figures and images for: Asymmetric oligomerization state and sequence patterning can tune multiphase condensate miscibility
Source: Nat Chem. 2024 Feb 21;16(7):1073–82. doi: 10.1038/s41557-024-01456-6 (PMC11230906; doi:10.1038/s41557-024-01456-6)

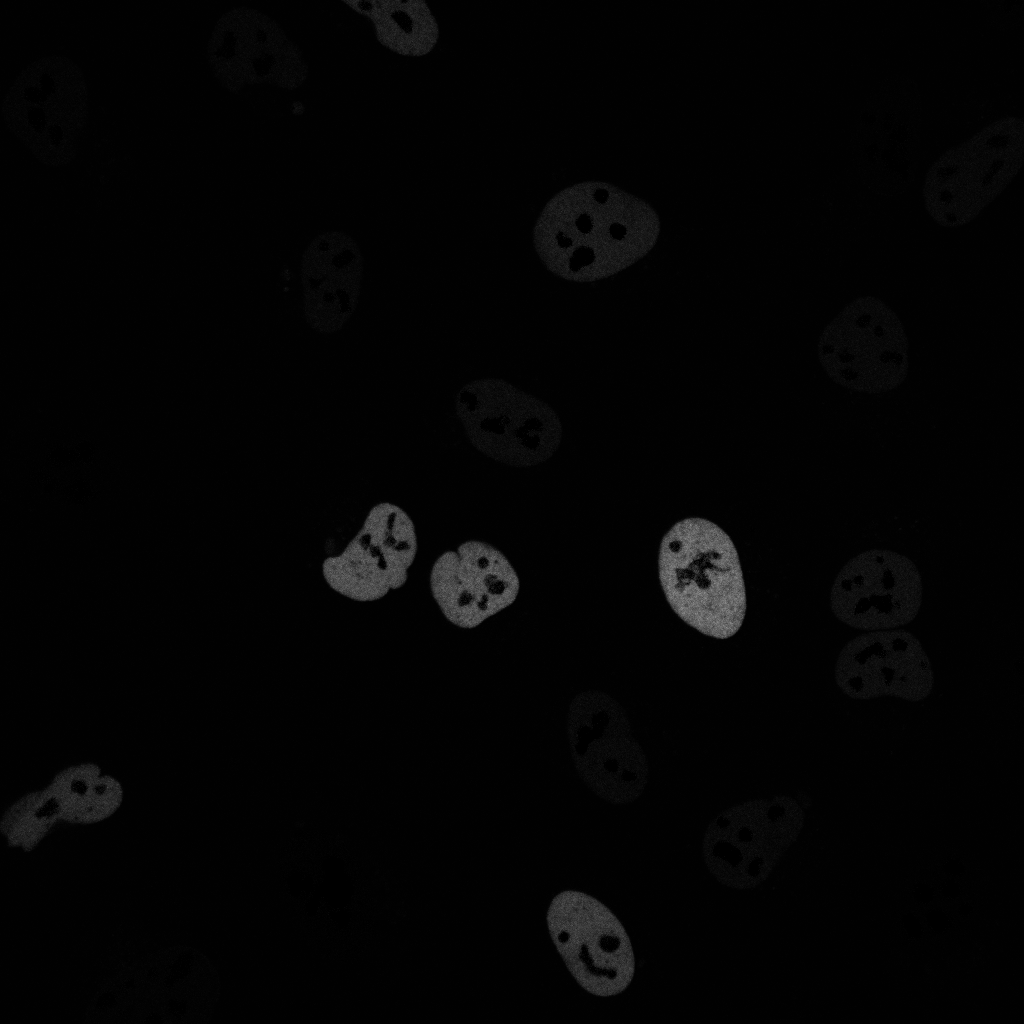

Supplement: Supplementary file 4 — Uncropped representative images. [file 41557_2024_1456_MOESM4_ESM.zip › iLID_FTH1_GFP_TimeSeries.tif]

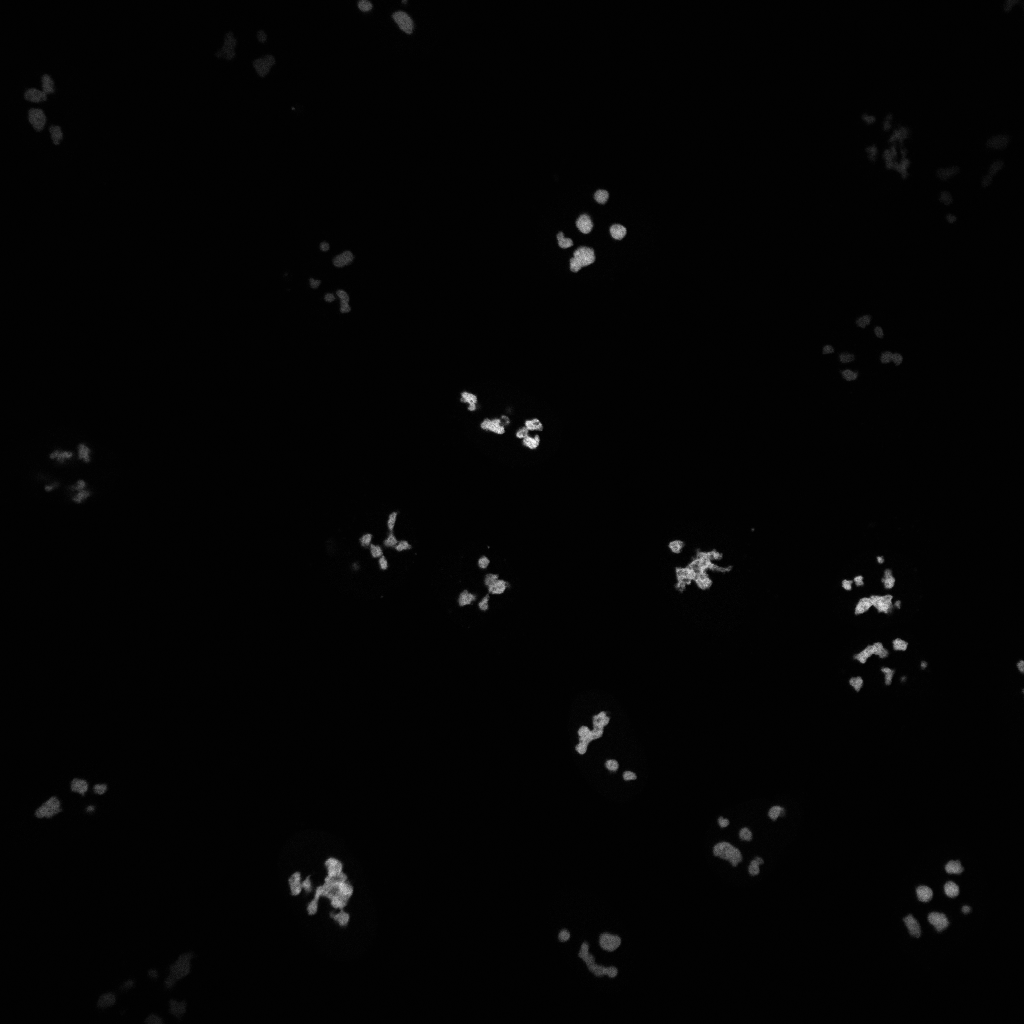

Supplement: Supplementary file 4 — Uncropped representative images. [file 41557_2024_1456_MOESM4_ESM.zip › NPM1_BFP_TimeSeries.tif]

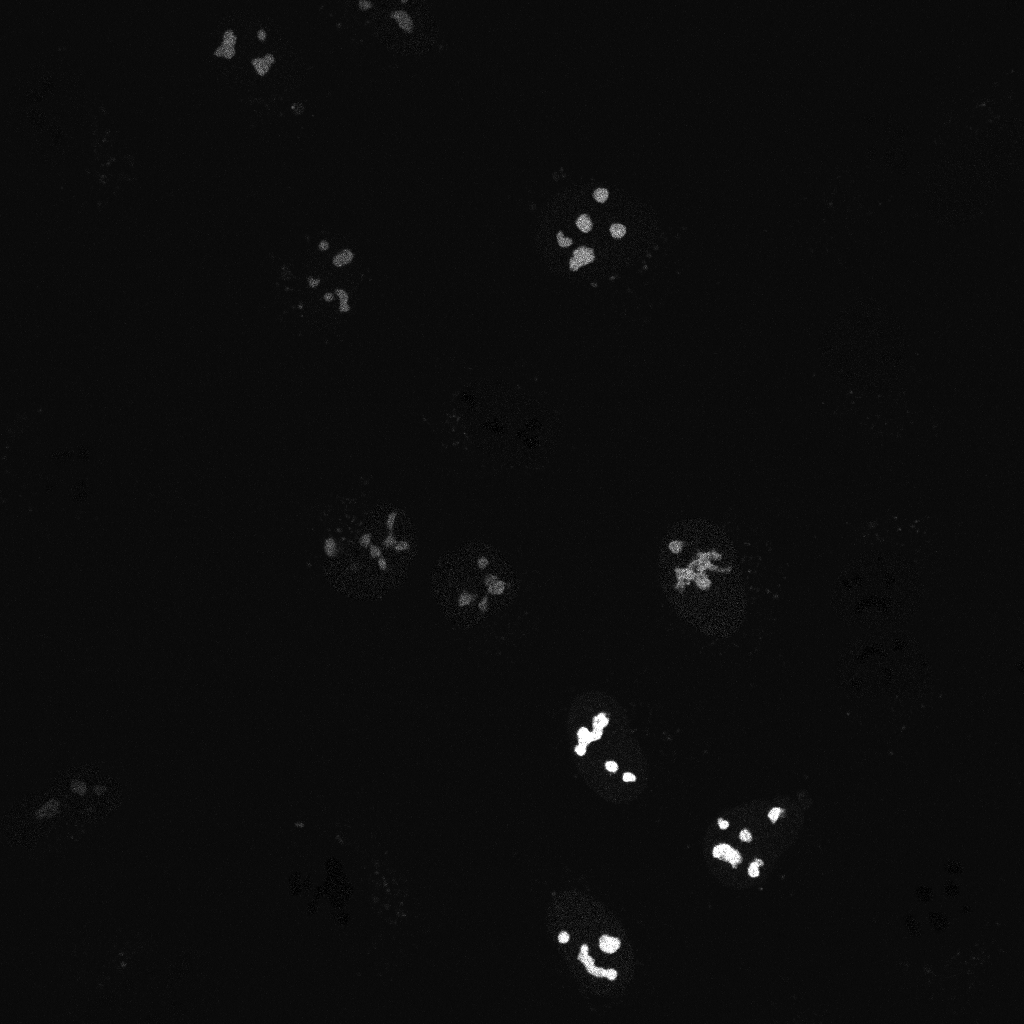

Supplement: Supplementary file 4 — Uncropped representative images. [file 41557_2024_1456_MOESM4_ESM.zip › NPM1_mCherry_SspB_TimeSeries.tif]

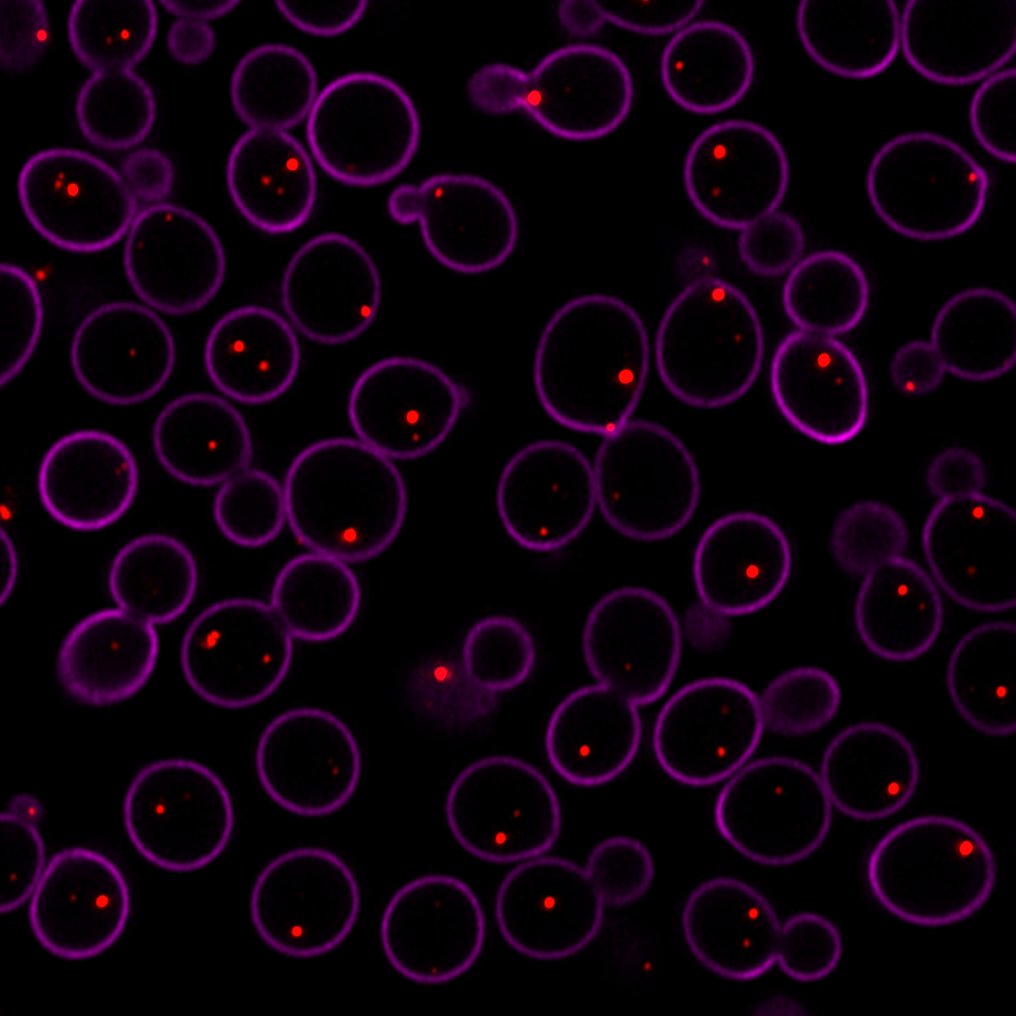

Supplement: Supplementary file 5 — Statistical source data. [file 41557_2024_1456_MOESM5_ESM.zip › Fig 3a_left.jpg]

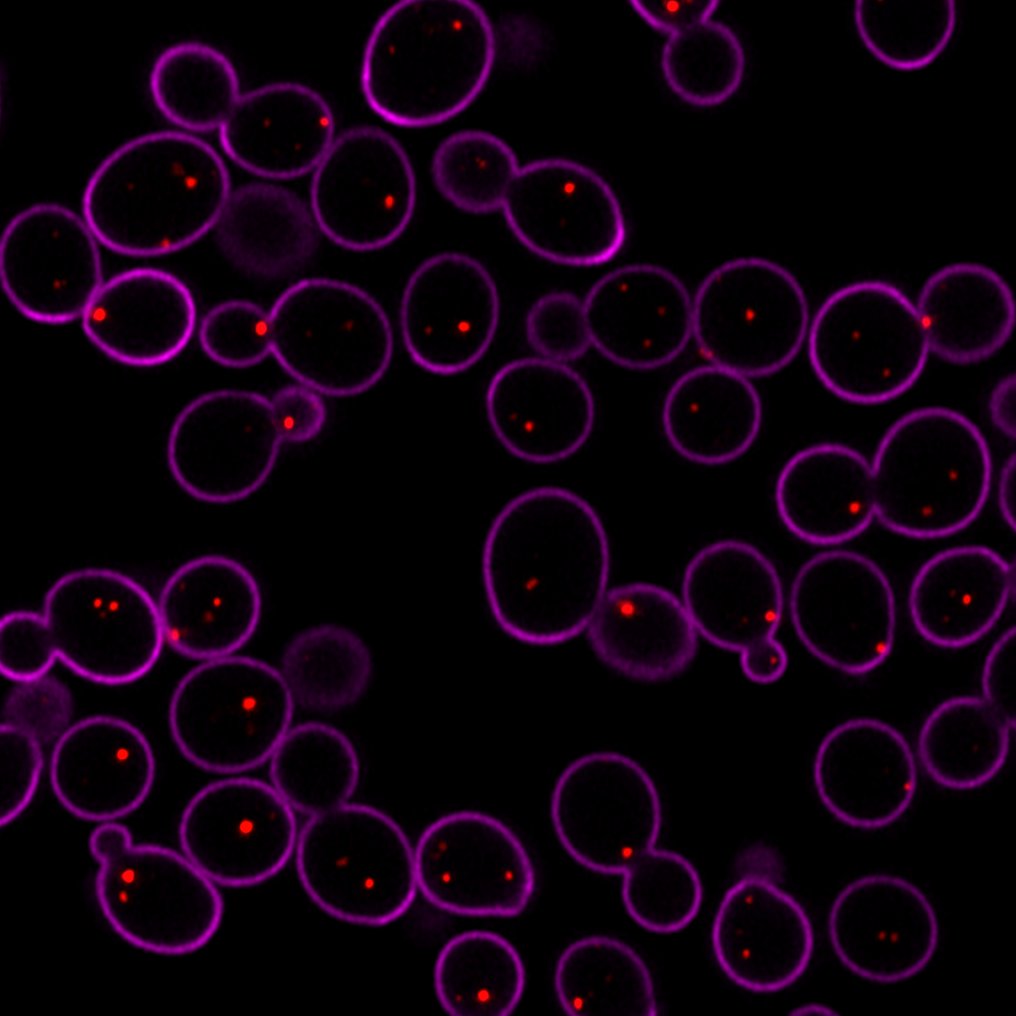

Supplement: Supplementary file 5 — Statistical source data. [file 41557_2024_1456_MOESM5_ESM.zip › Fig 3a_middle.jpg]

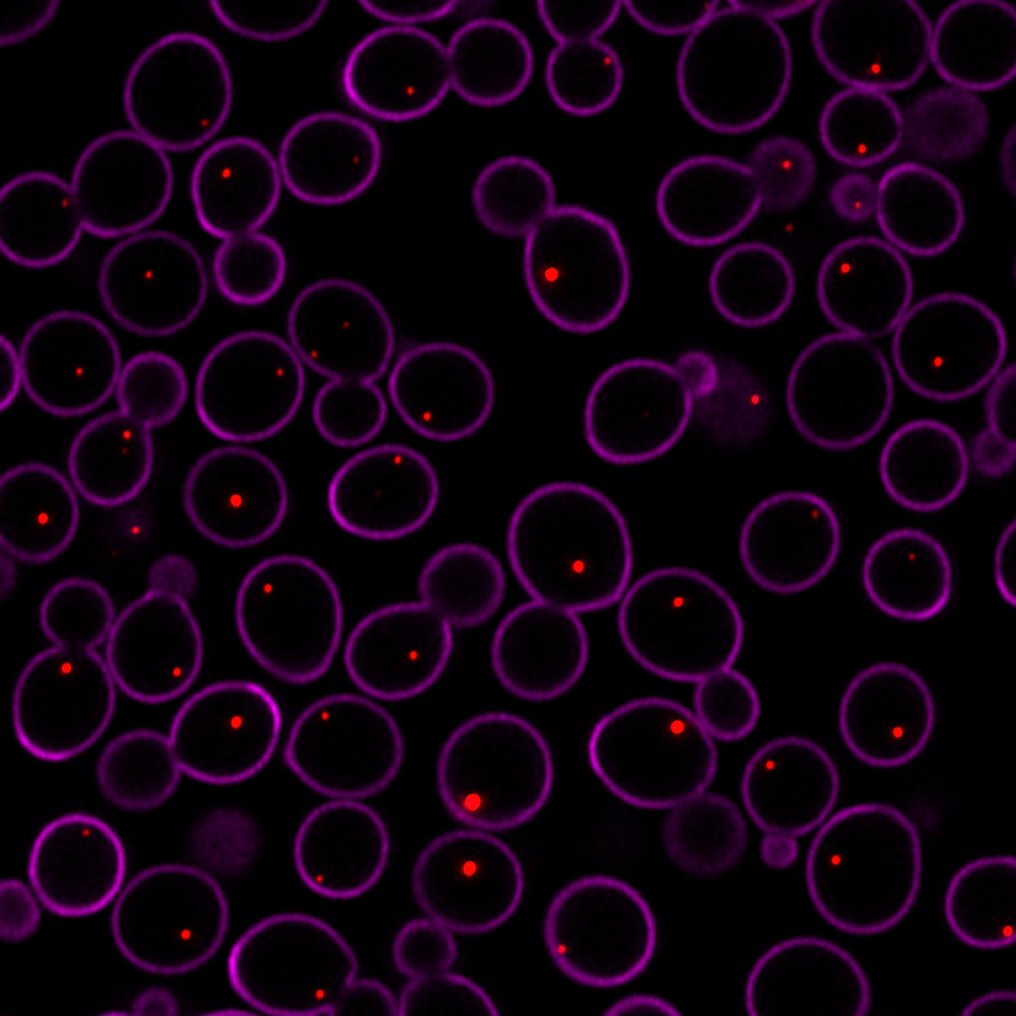

Supplement: Supplementary file 5 — Statistical source data. [file 41557_2024_1456_MOESM5_ESM.zip › Fig 3a_right.jpg]

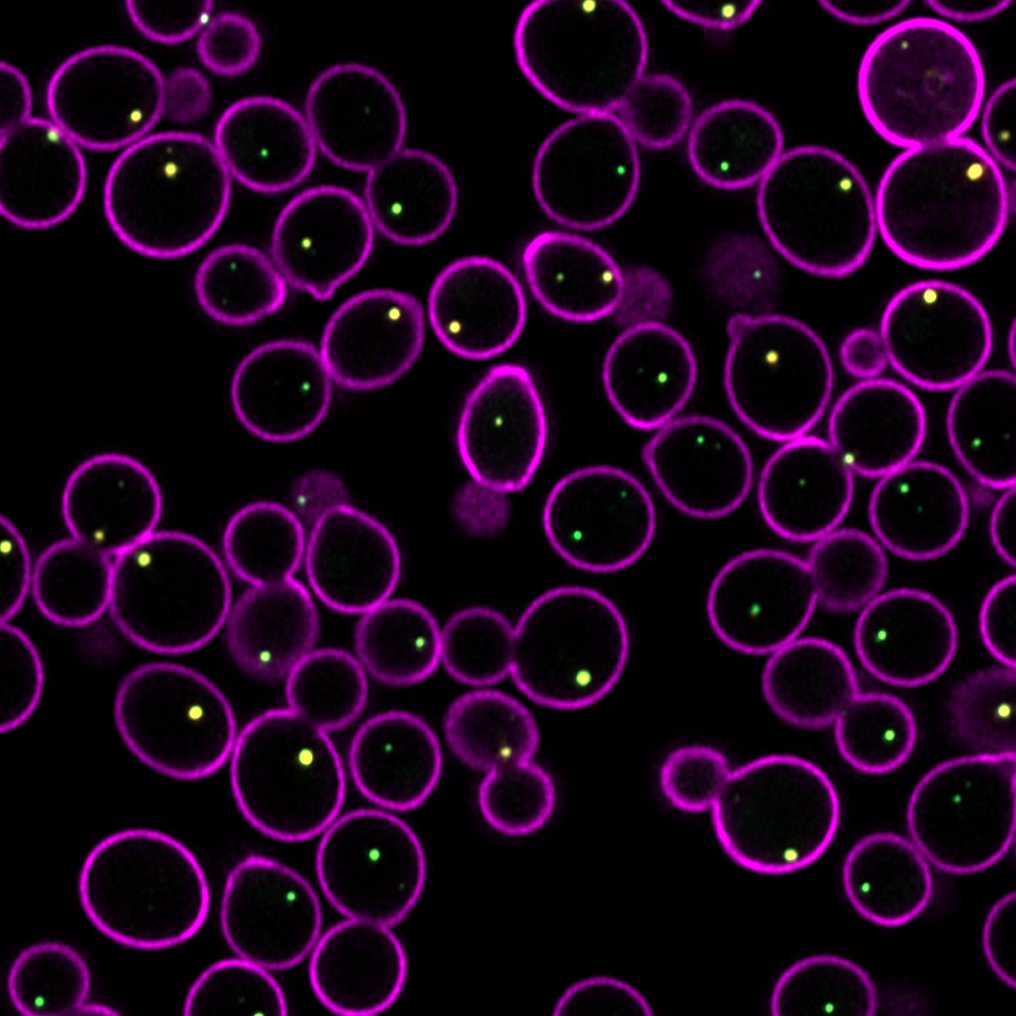

Supplement: Supplementary file 5 — Statistical source data. [file 41557_2024_1456_MOESM5_ESM.zip › Fig 3c_left.jpg]

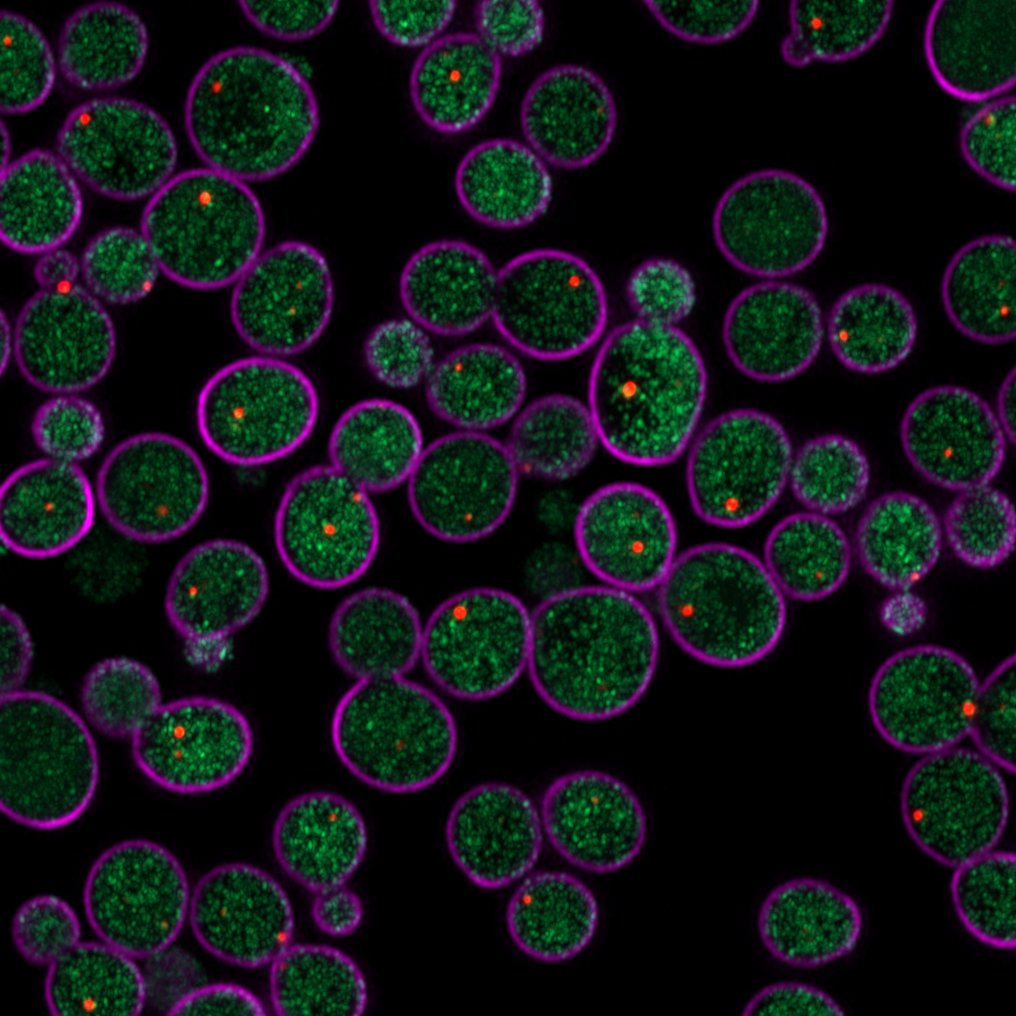

Supplement: Supplementary file 5 — Statistical source data. [file 41557_2024_1456_MOESM5_ESM.zip › Fig 3c_middle.jpg]

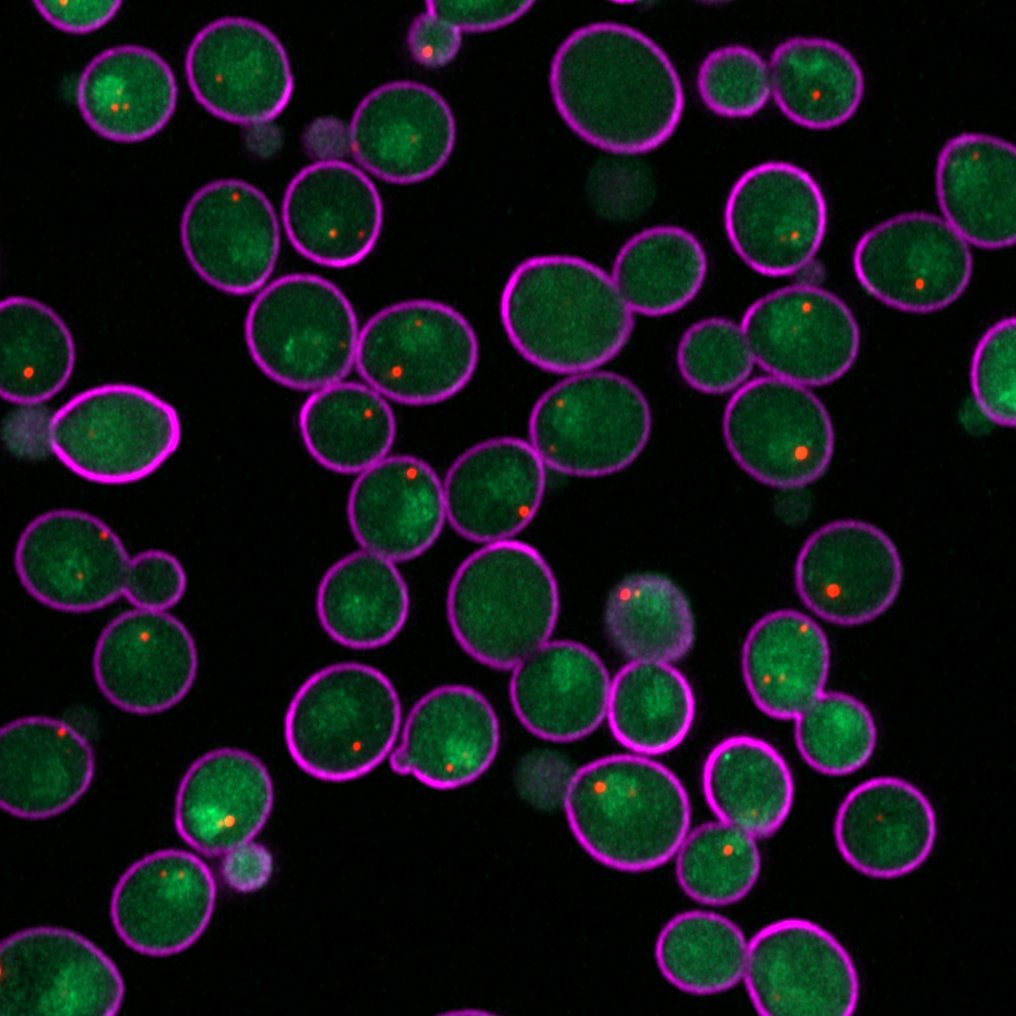

Supplement: Supplementary file 5 — Statistical source data. [file 41557_2024_1456_MOESM5_ESM.zip › Fig 3c_right.jpg]

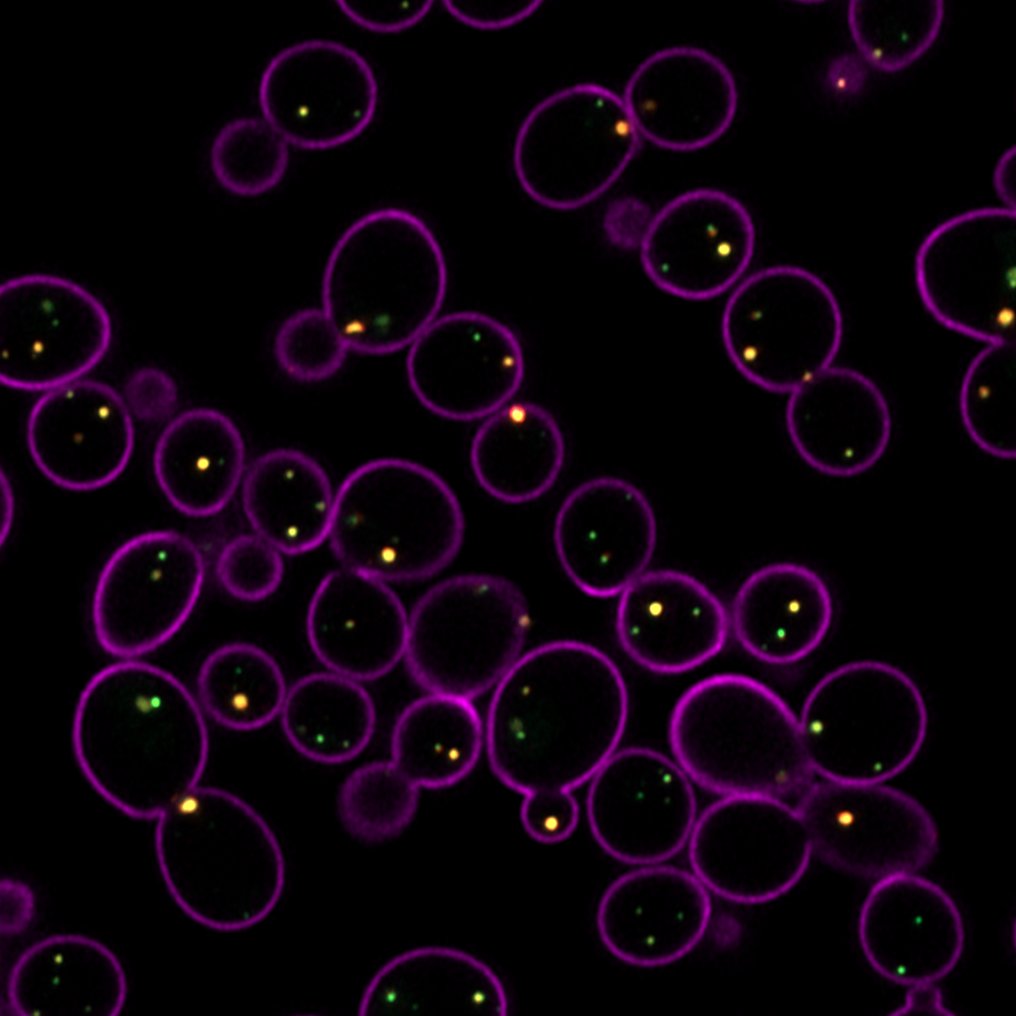

Supplement: Supplementary file 5 — Statistical source data. [file 41557_2024_1456_MOESM5_ESM.zip › Fig 3e_left.jpg]

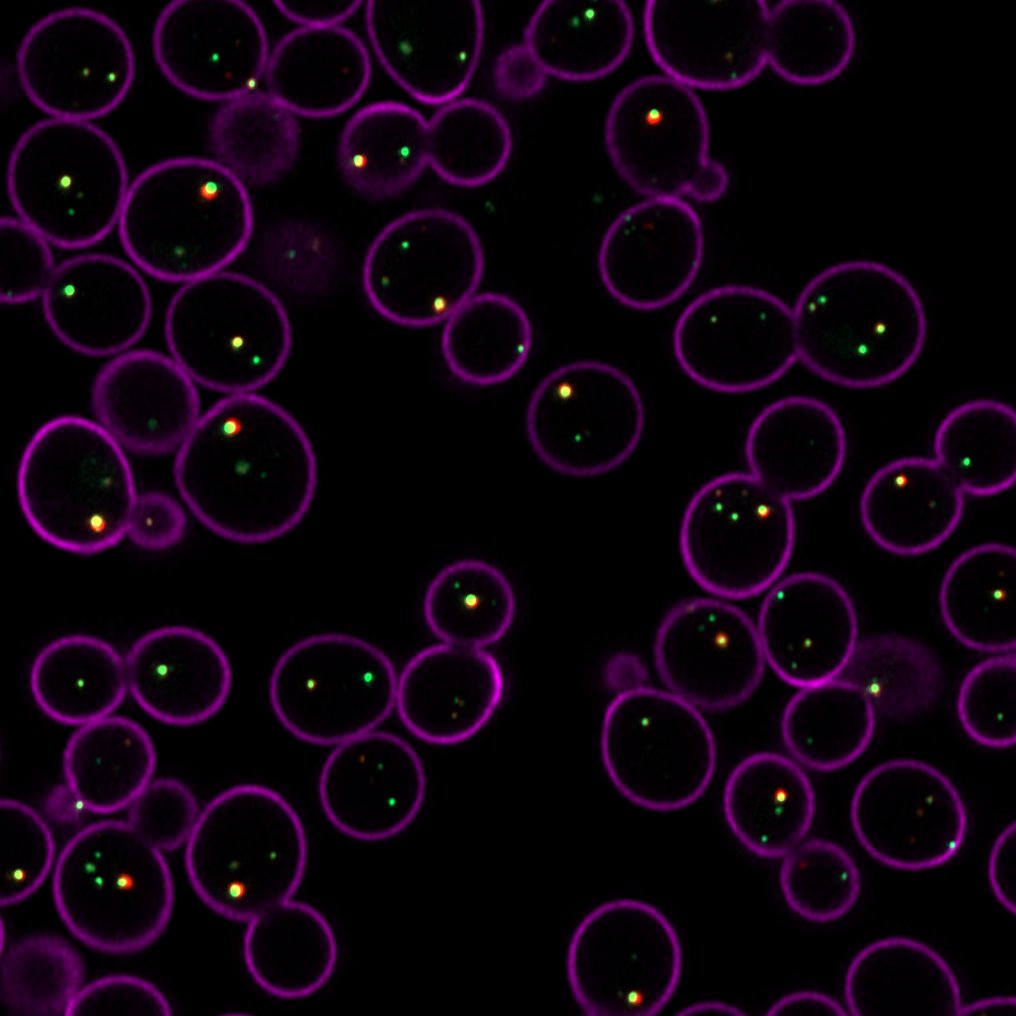

Supplement: Supplementary file 5 — Statistical source data. [file 41557_2024_1456_MOESM5_ESM.zip › Fig 3e_right.jpg]

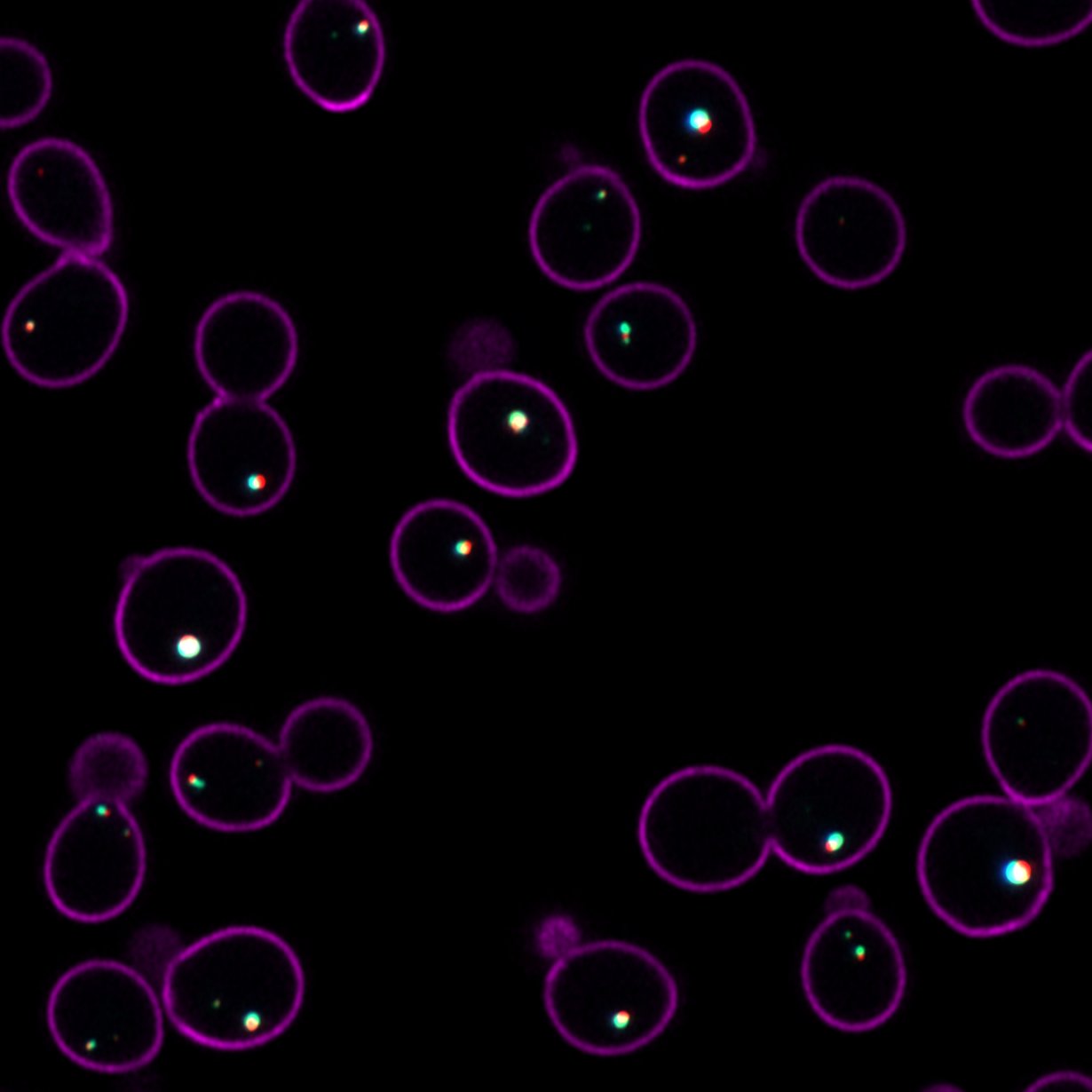

Supplement: Supplementary file 6 — Statistical source data. [file 41557_2024_1456_MOESM6_ESM.zip › Fig 4b_full hnRNPA1c valency_merge.jpg]

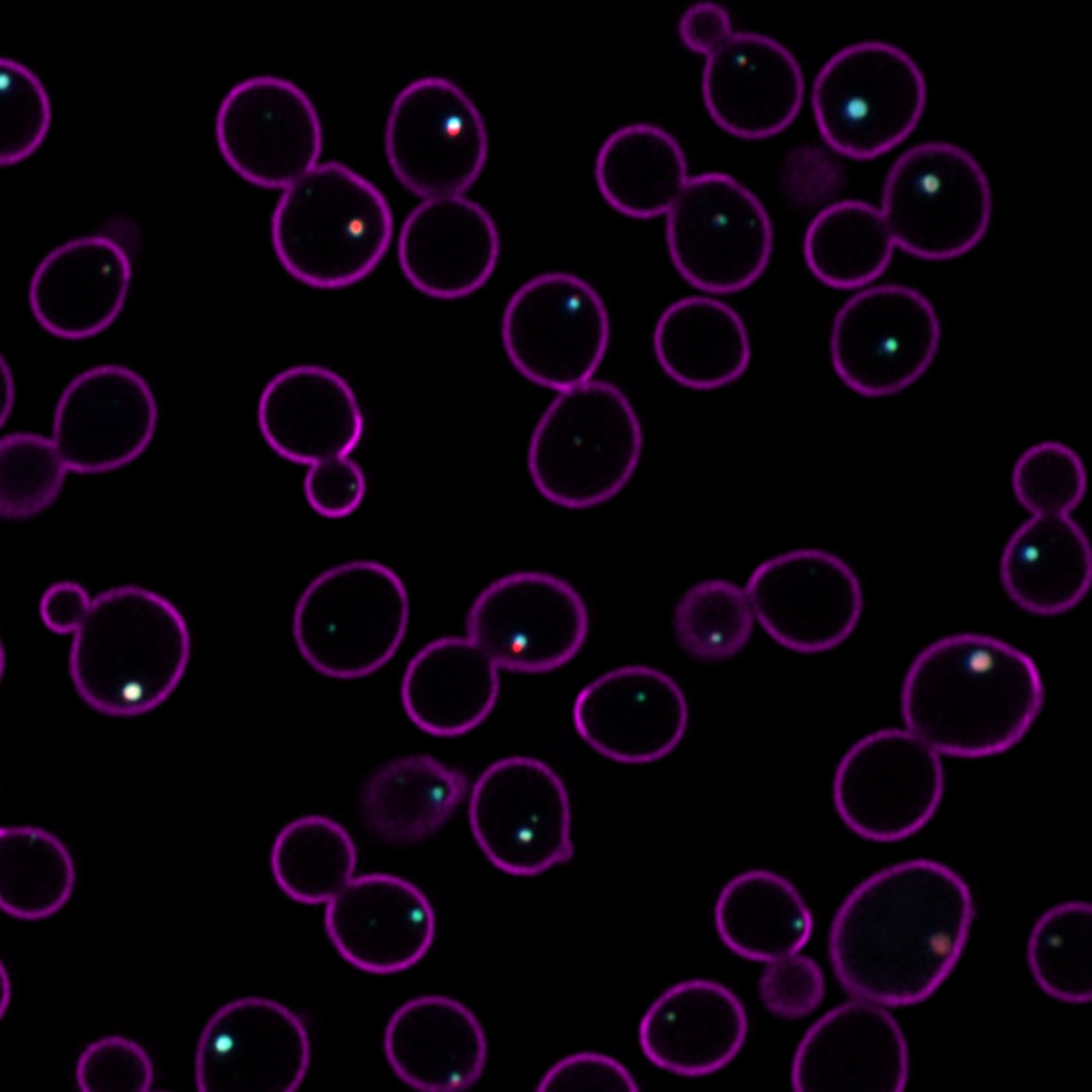

Supplement: Supplementary file 6 — Statistical source data. [file 41557_2024_1456_MOESM6_ESM.zip › Fig 4b_high hnRNPA1c valency_merge.jpg]

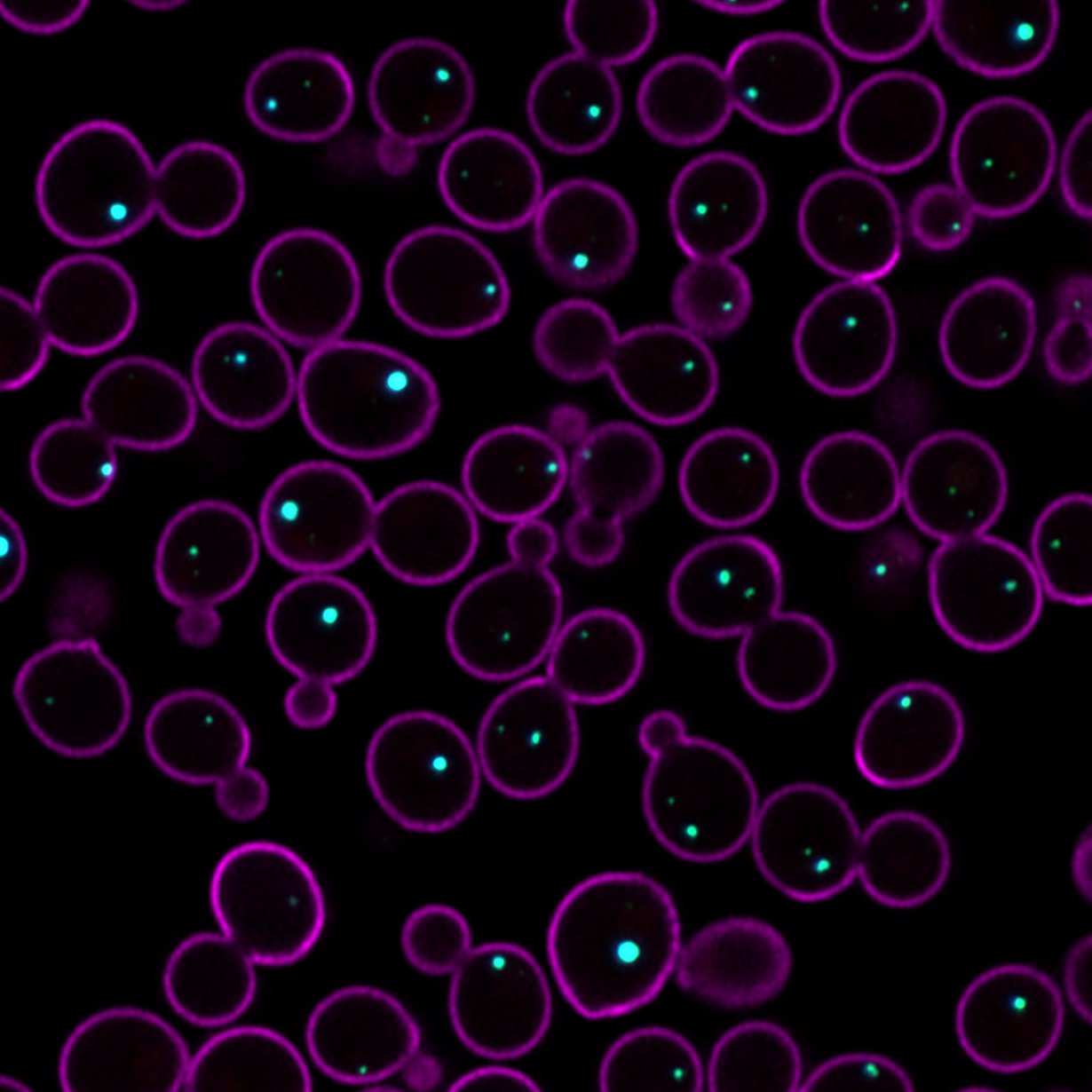

Supplement: Supplementary file 6 — Statistical source data. [file 41557_2024_1456_MOESM6_ESM.zip › Fig 4b_low hnRNPA1c valency_merge.jpg]

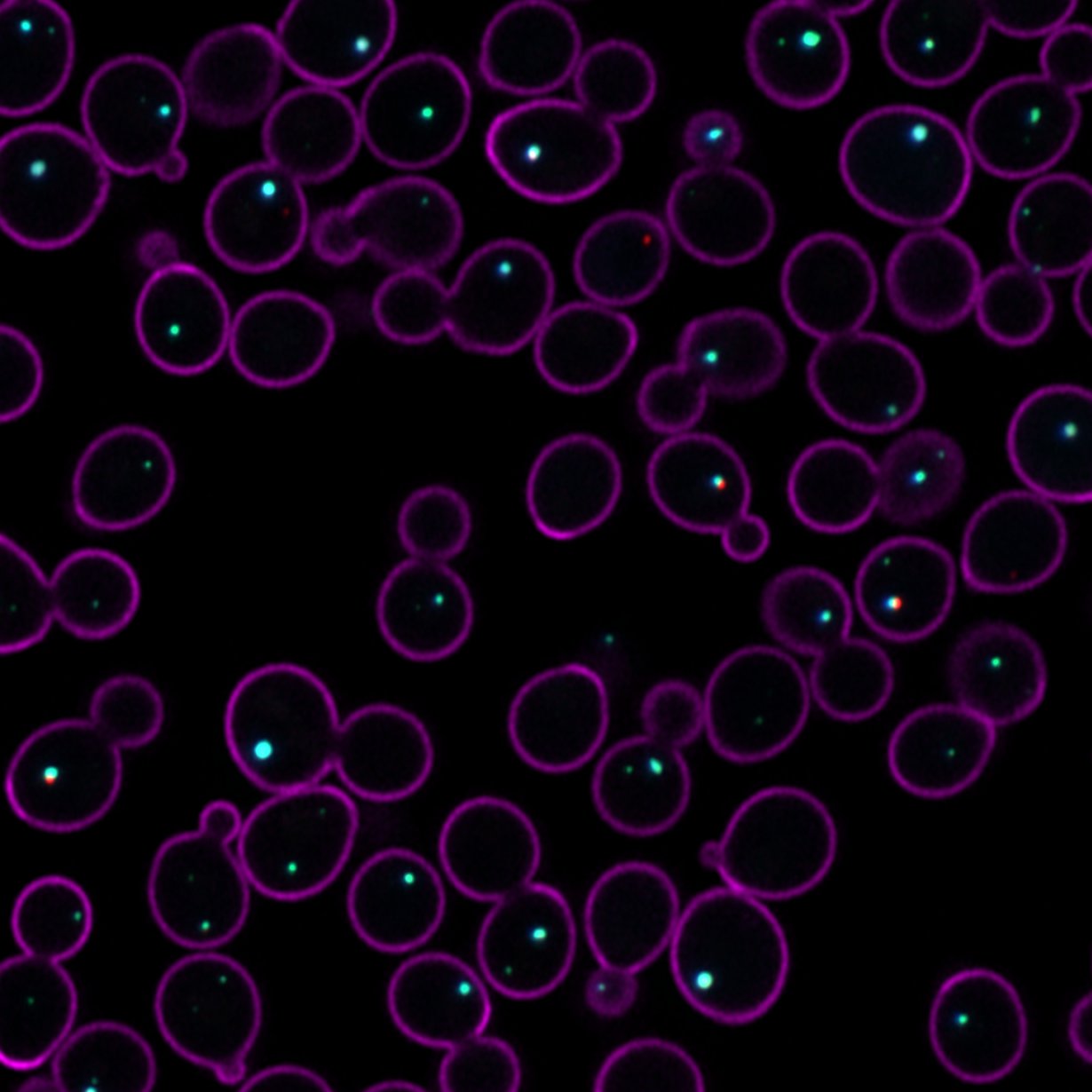

Supplement: Supplementary file 6 — Statistical source data. [file 41557_2024_1456_MOESM6_ESM.zip › Fig 4b_medium hnRNPA1c valency_merge.jpg]

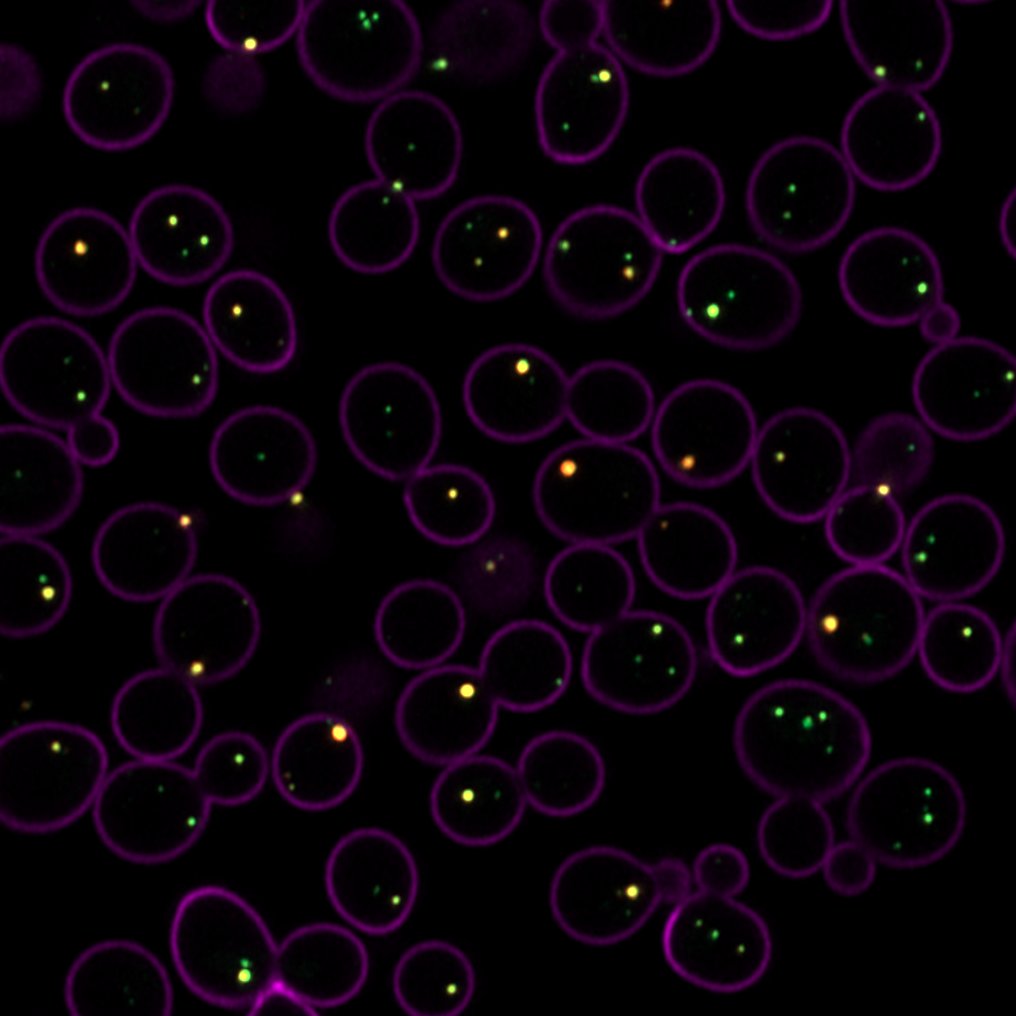

Supplement: Supplementary file 7 — Statistical source data. [file 41557_2024_1456_MOESM7_ESM.zip › Fig5a_bottom_left_yKX294.jpg]

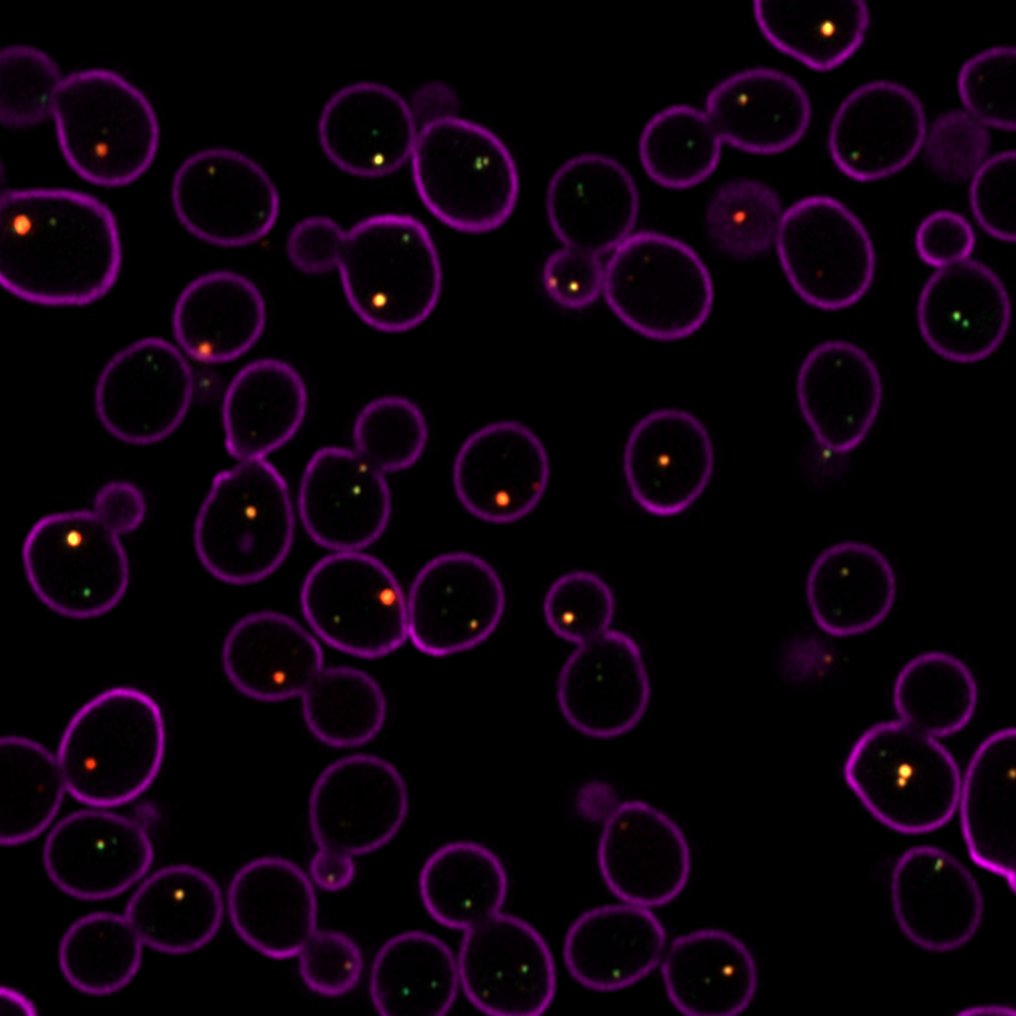

Supplement: Supplementary file 7 — Statistical source data. [file 41557_2024_1456_MOESM7_ESM.zip › Fig5a_bottom_middle_yKX297.jpg]

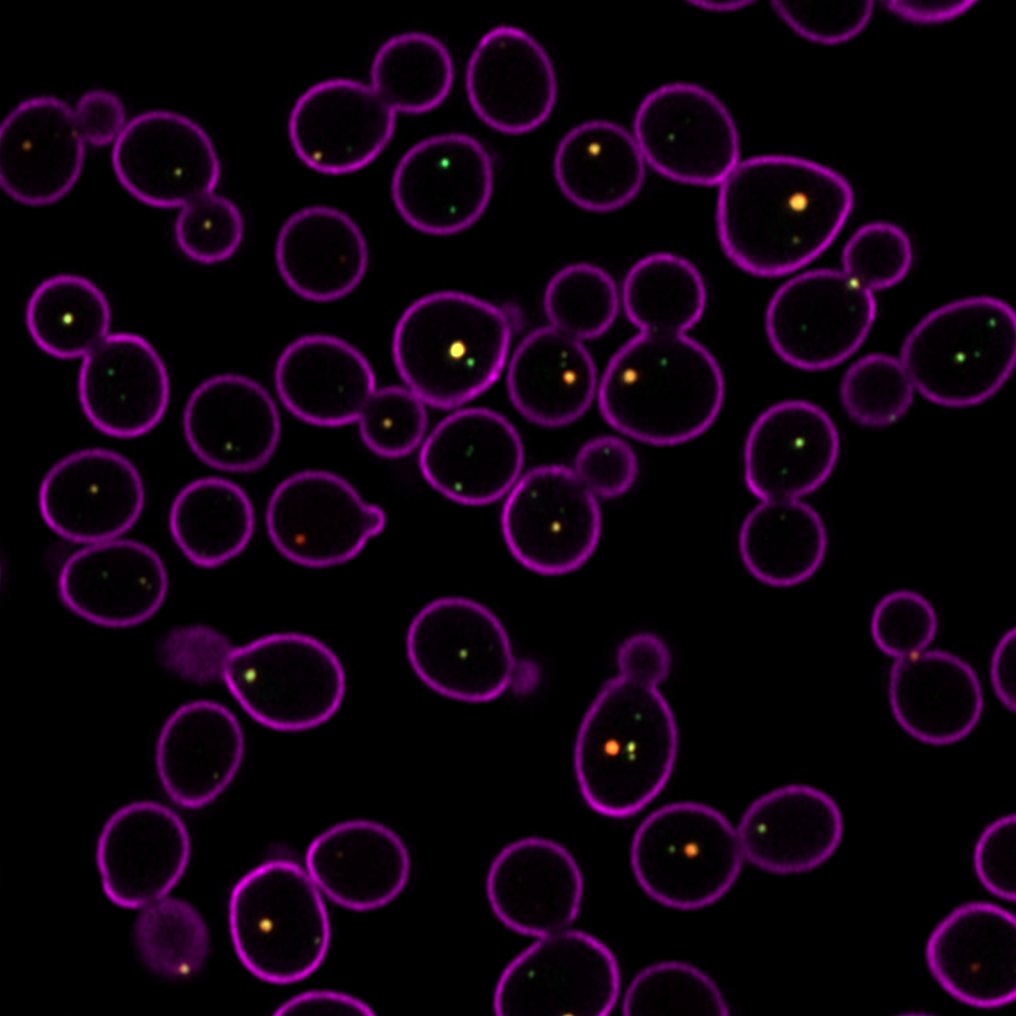

Supplement: Supplementary file 7 — Statistical source data. [file 41557_2024_1456_MOESM7_ESM.zip › Fig5a_bottom_right_yKX331.jpg]

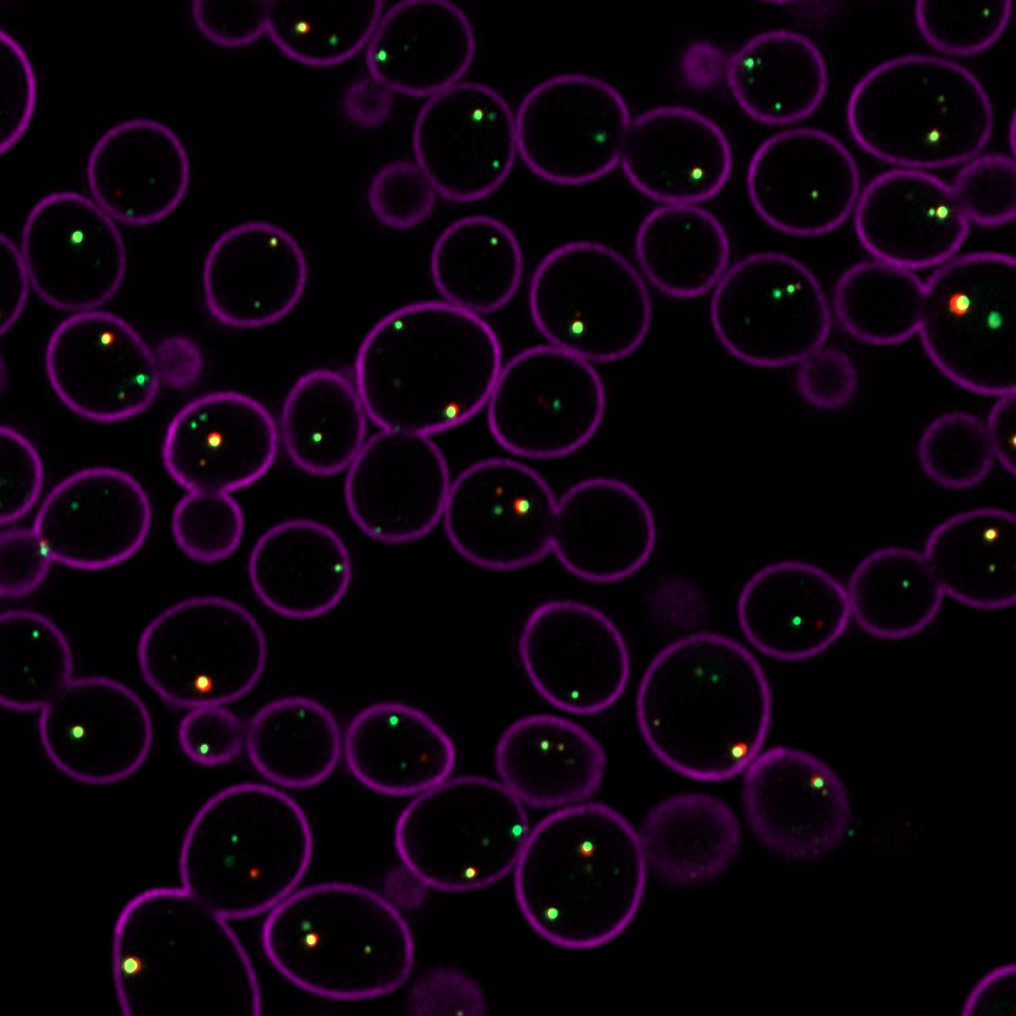

Supplement: Supplementary file 7 — Statistical source data. [file 41557_2024_1456_MOESM7_ESM.zip › Fig5a_middle_left_yKX173.jpg]

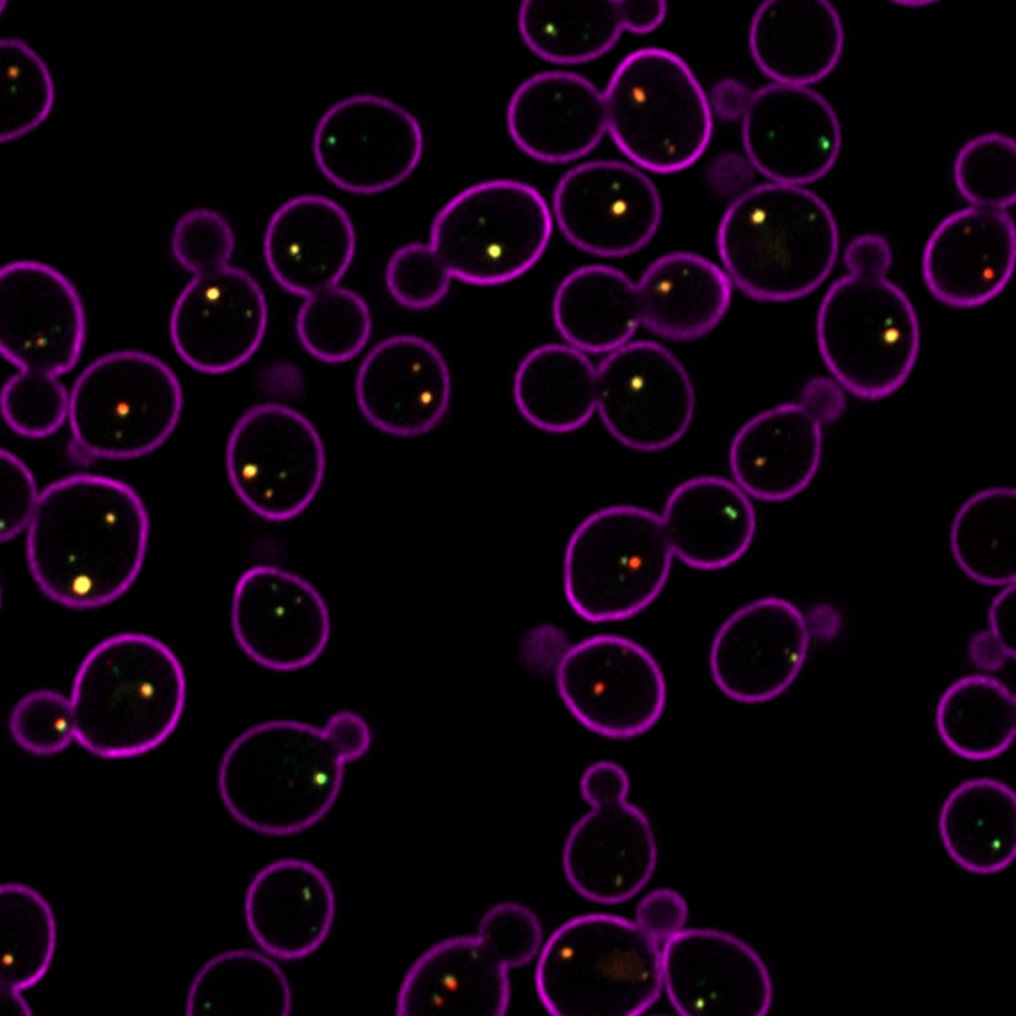

Supplement: Supplementary file 7 — Statistical source data. [file 41557_2024_1456_MOESM7_ESM.zip › Fig5a_middle_middle_yKX176.jpg]

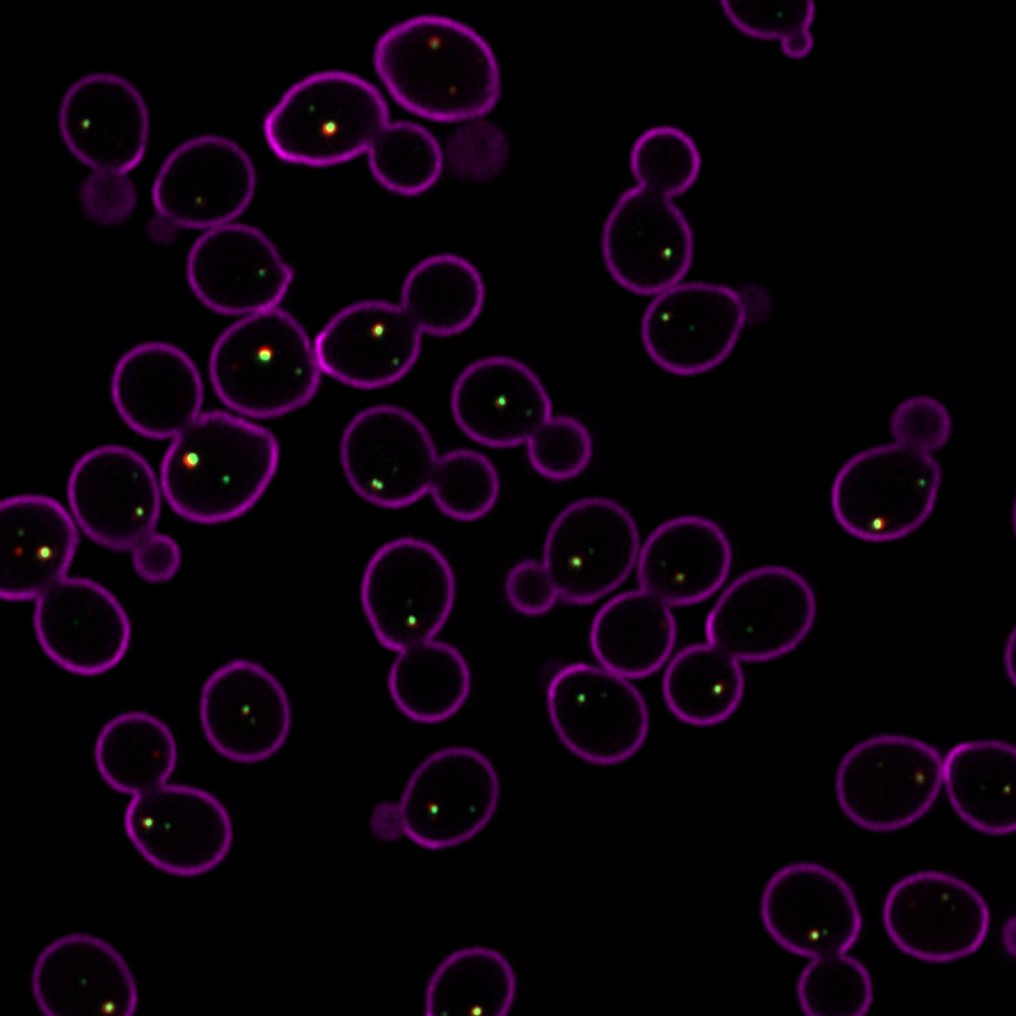

Supplement: Supplementary file 7 — Statistical source data. [file 41557_2024_1456_MOESM7_ESM.zip › Fig5a_middle_right_yKX332.jpg]

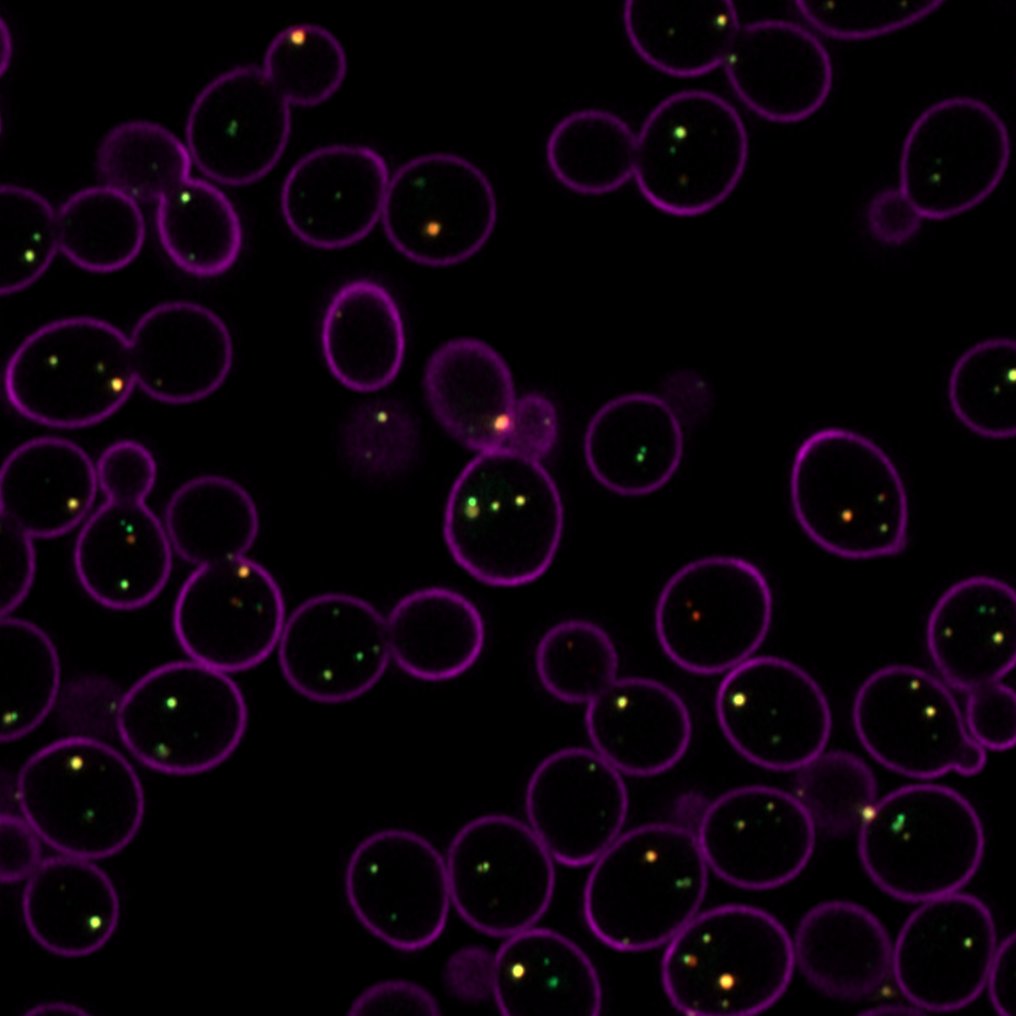

Supplement: Supplementary file 7 — Statistical source data. [file 41557_2024_1456_MOESM7_ESM.zip › Fig5a_top_left_yKX172.jpg]

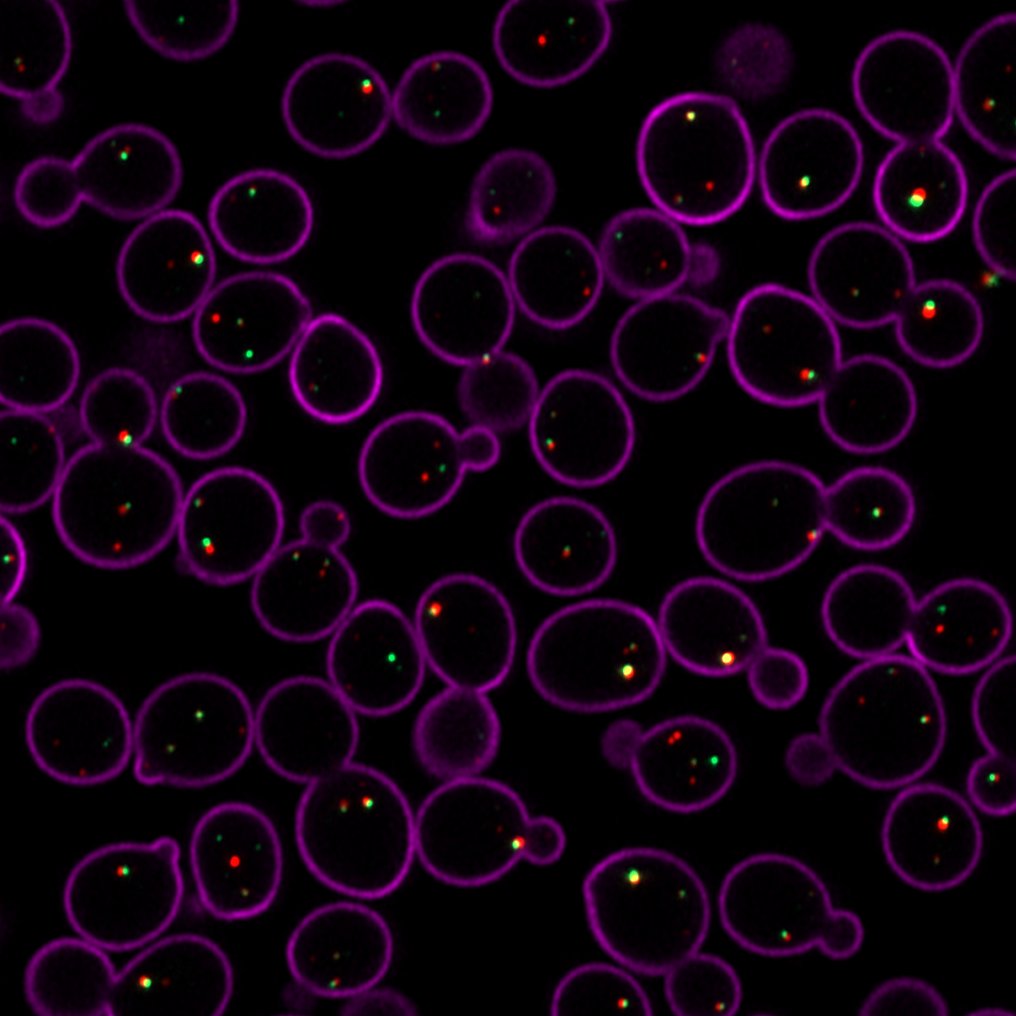

Supplement: Supplementary file 7 — Statistical source data. [file 41557_2024_1456_MOESM7_ESM.zip › Fig5a_top_middle_yKX175.jpg]

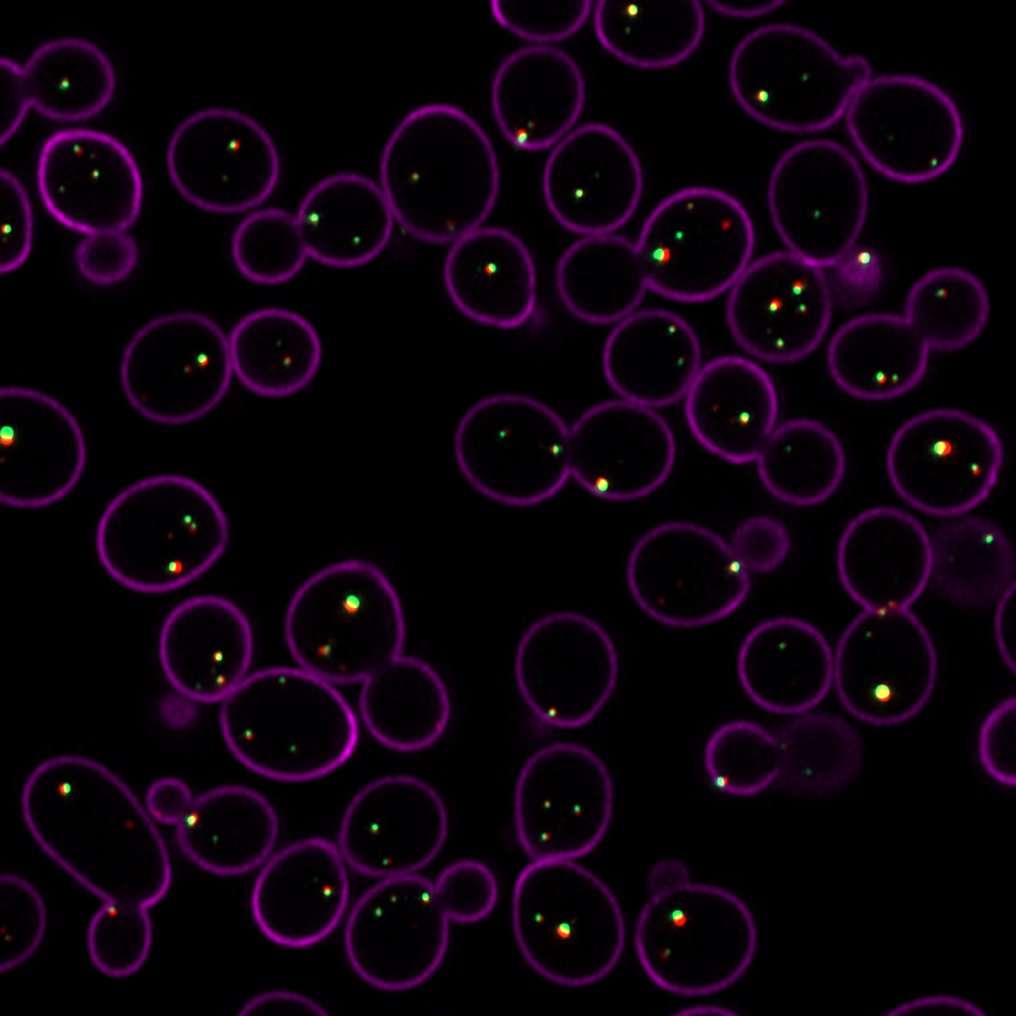

Supplement: Supplementary file 7 — Statistical source data. [file 41557_2024_1456_MOESM7_ESM.zip › Fig5a_top_right_yKX296.jpg]

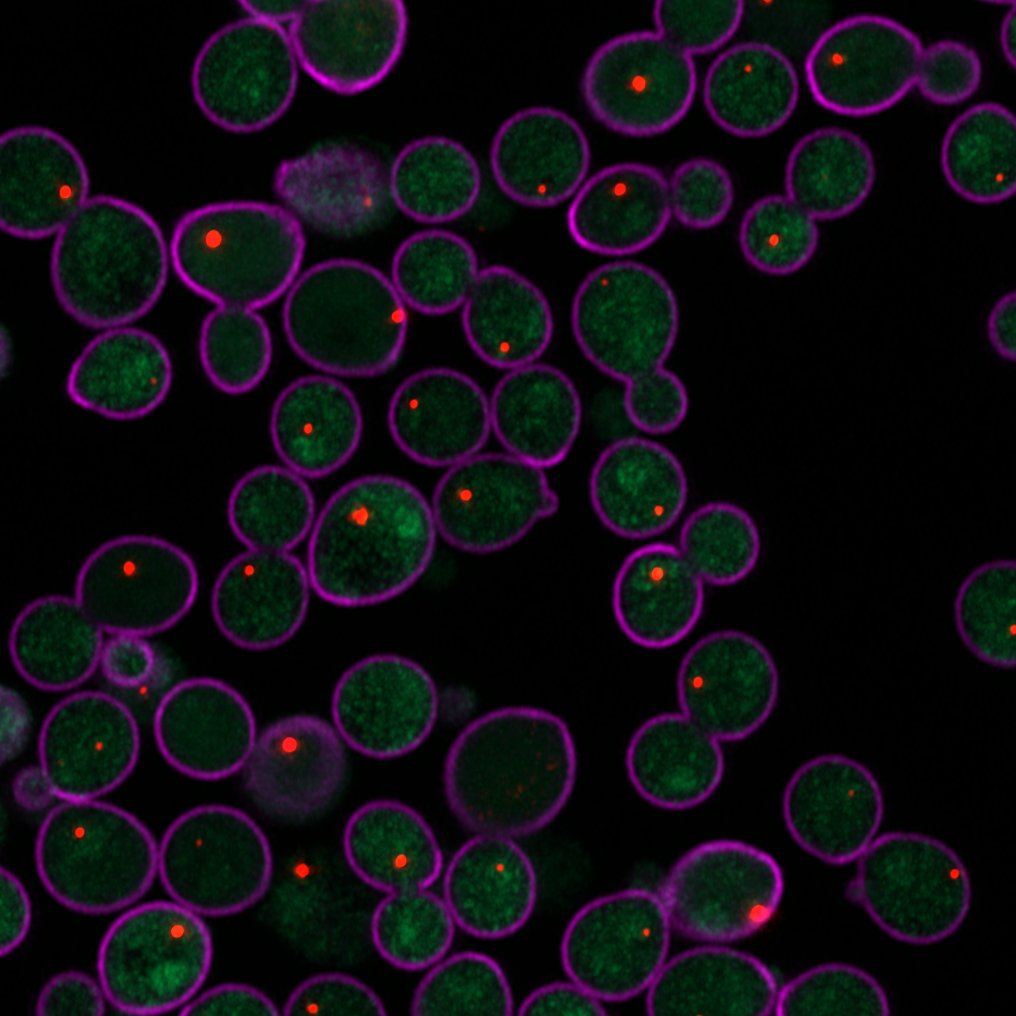

Supplement: Supplementary file 10 — Statistical source data. [file 41557_2024_1456_MOESM10_ESM.zip › Extended Dara Fig2a_bottom_left_yKX377.jpg]

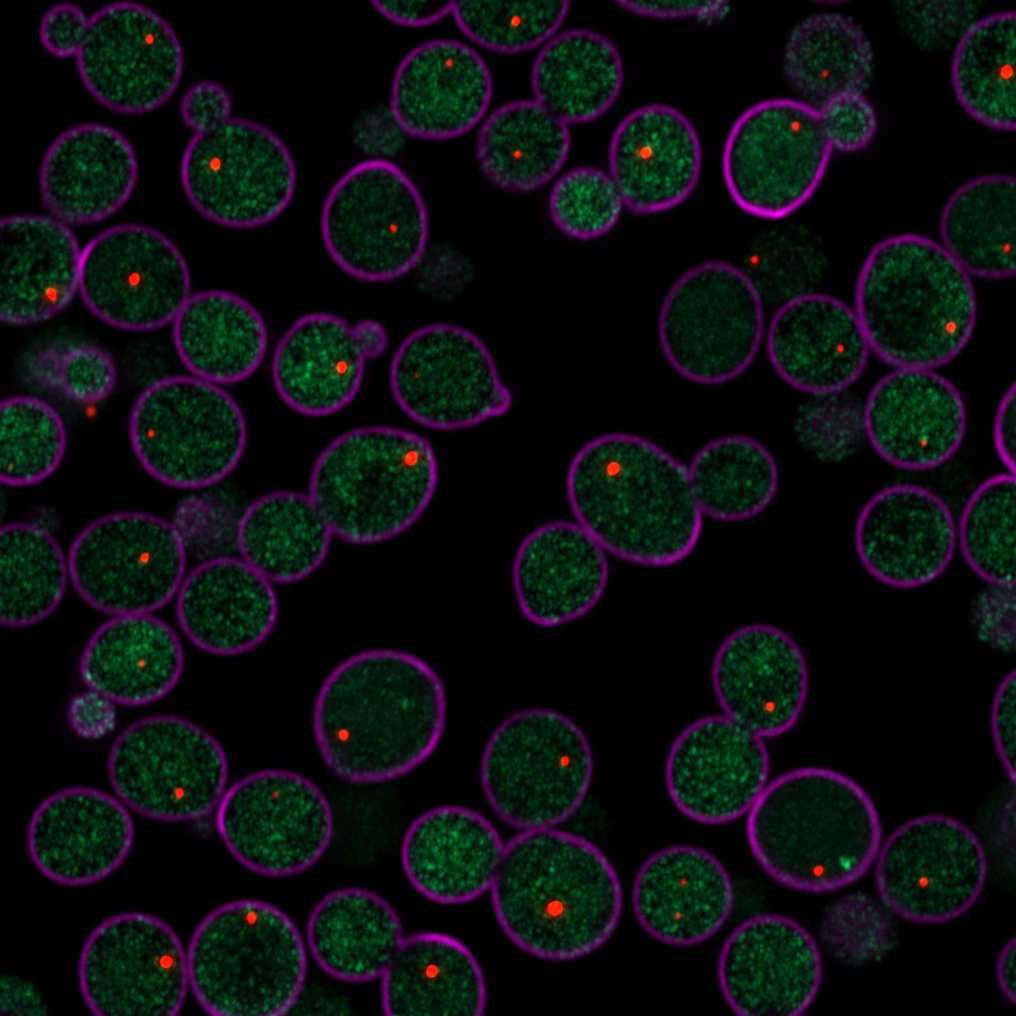

Supplement: Supplementary file 10 — Statistical source data. [file 41557_2024_1456_MOESM10_ESM.zip › Extended Dara Fig2a_bottom_middle_yKX378.jpg]

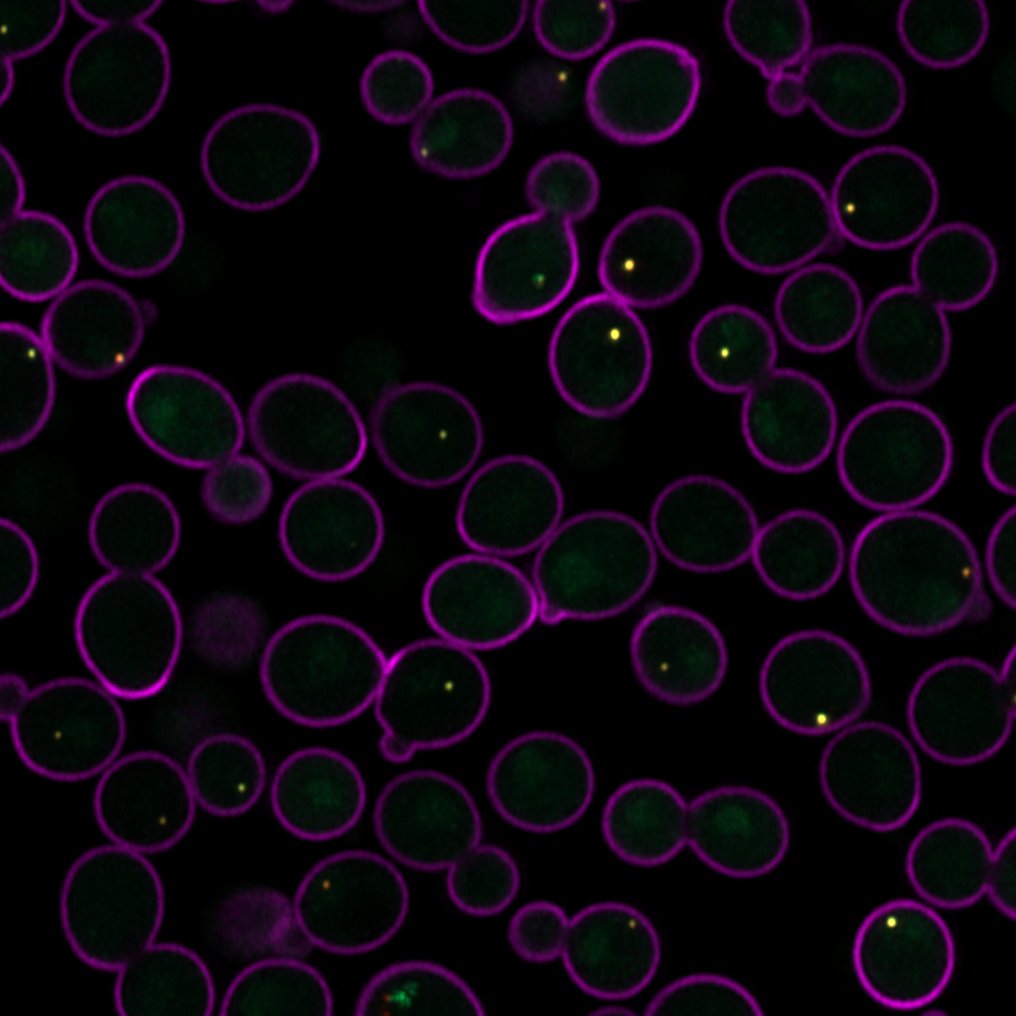

Supplement: Supplementary file 10 — Statistical source data. [file 41557_2024_1456_MOESM10_ESM.zip › Extended Dara Fig2a_bottom_right_yKX379.jpg]

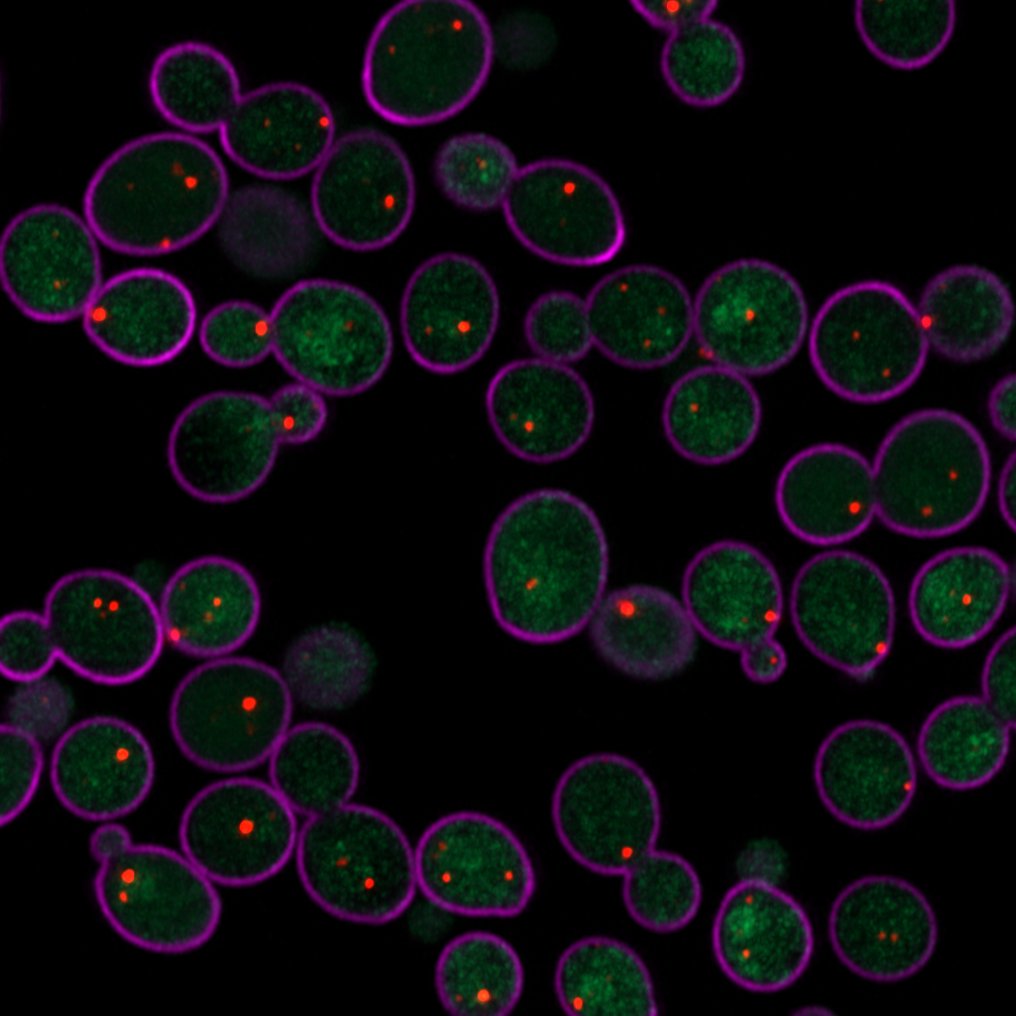

Supplement: Supplementary file 10 — Statistical source data. [file 41557_2024_1456_MOESM10_ESM.zip › Extended Dara Fig2a_middle_left_yKX374.jpg]

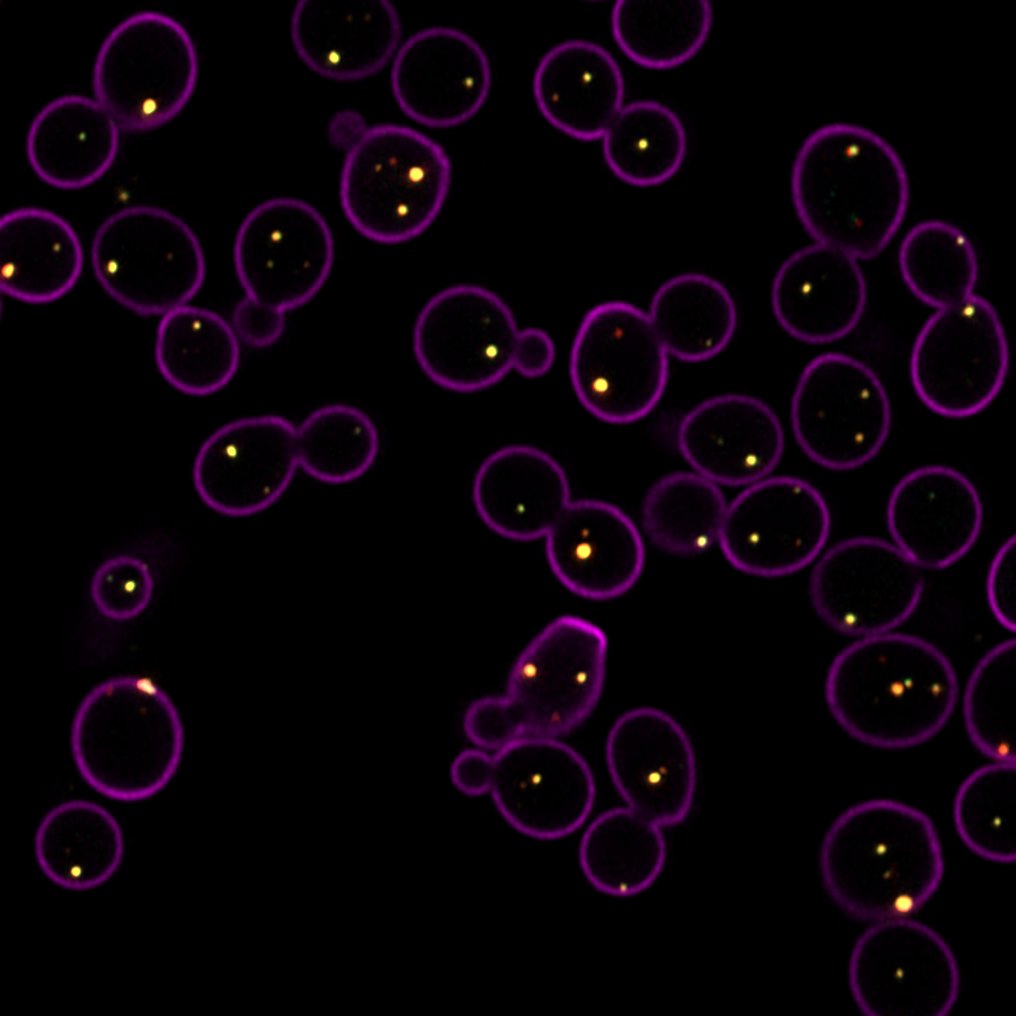

Supplement: Supplementary file 10 — Statistical source data. [file 41557_2024_1456_MOESM10_ESM.zip › Extended Dara Fig2a_middle_middle_yKX375.jpg]

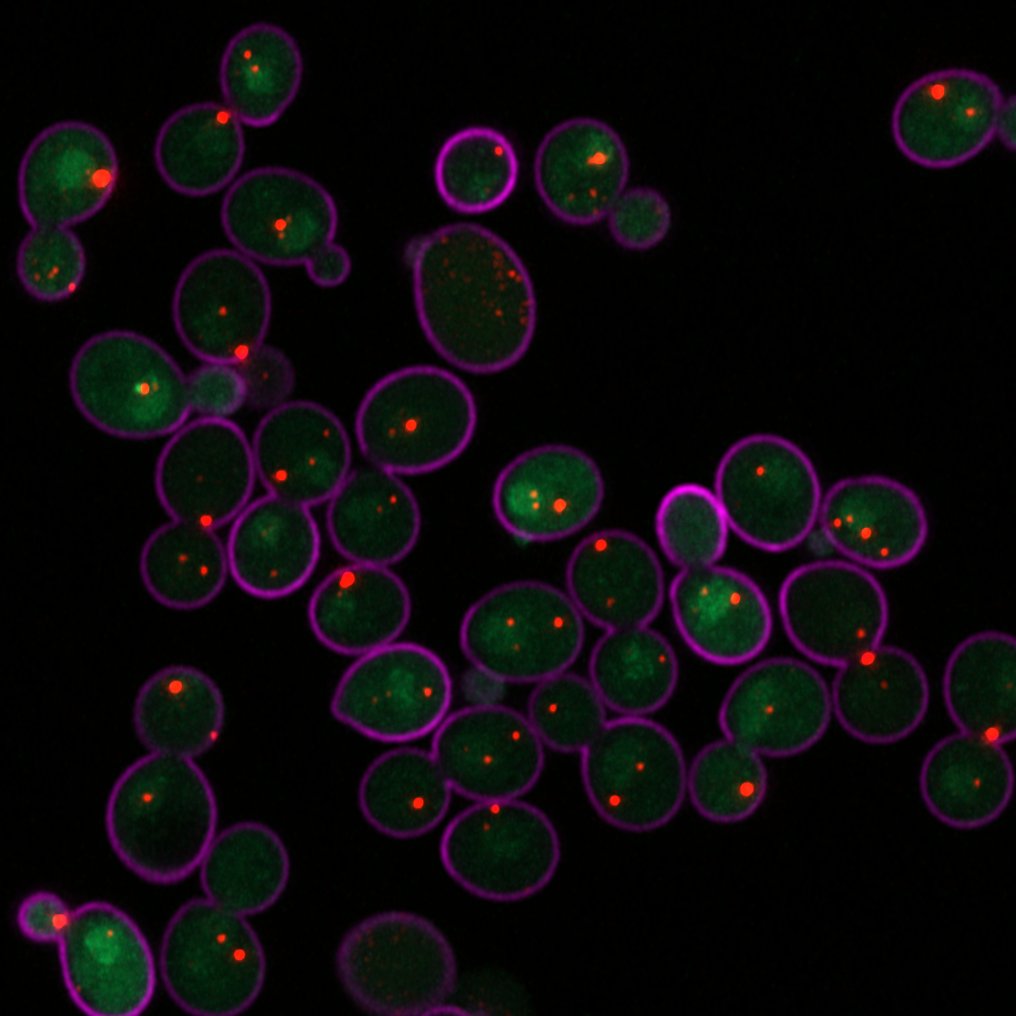

Supplement: Supplementary file 10 — Statistical source data. [file 41557_2024_1456_MOESM10_ESM.zip › Extended Dara Fig2a_middle_right_yKX376.jpg]

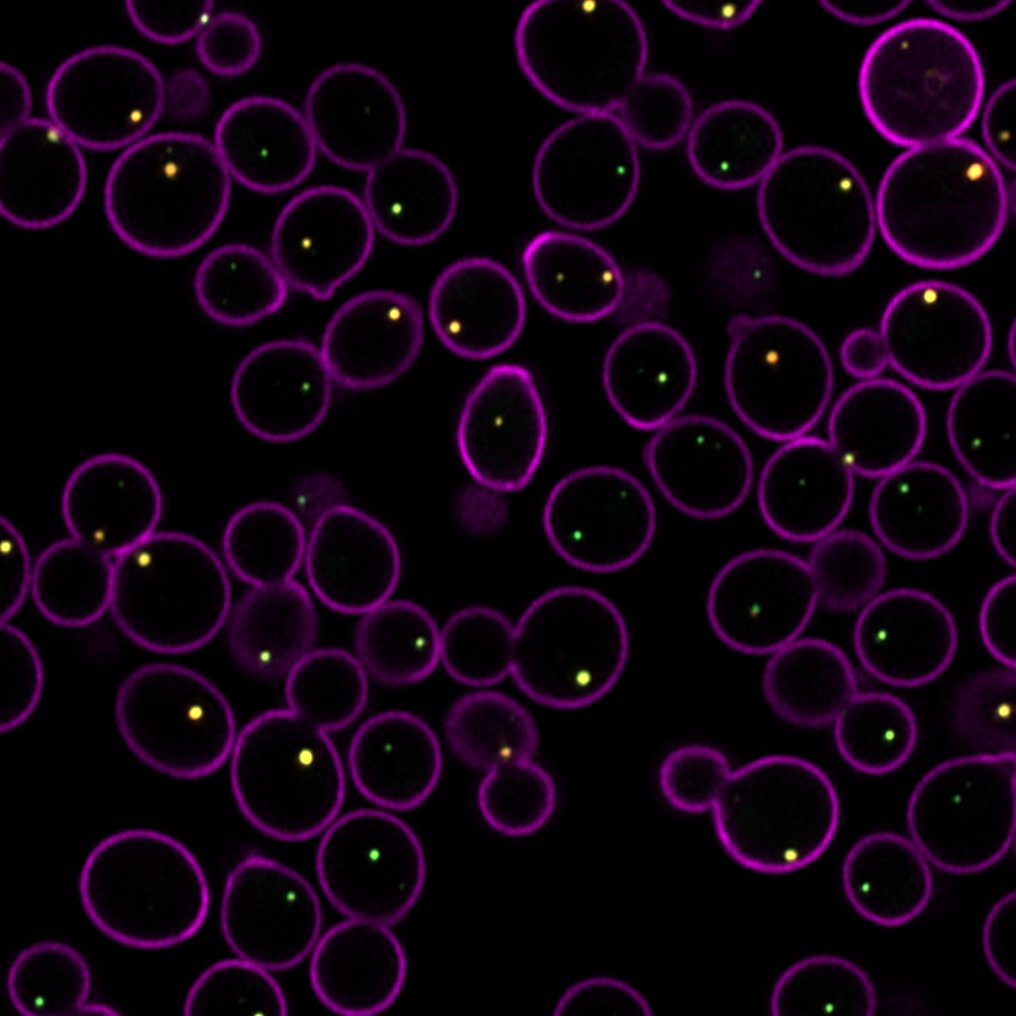

Supplement: Supplementary file 10 — Statistical source data. [file 41557_2024_1456_MOESM10_ESM.zip › Extended Dara Fig2a_top_left_yKX371.jpg]

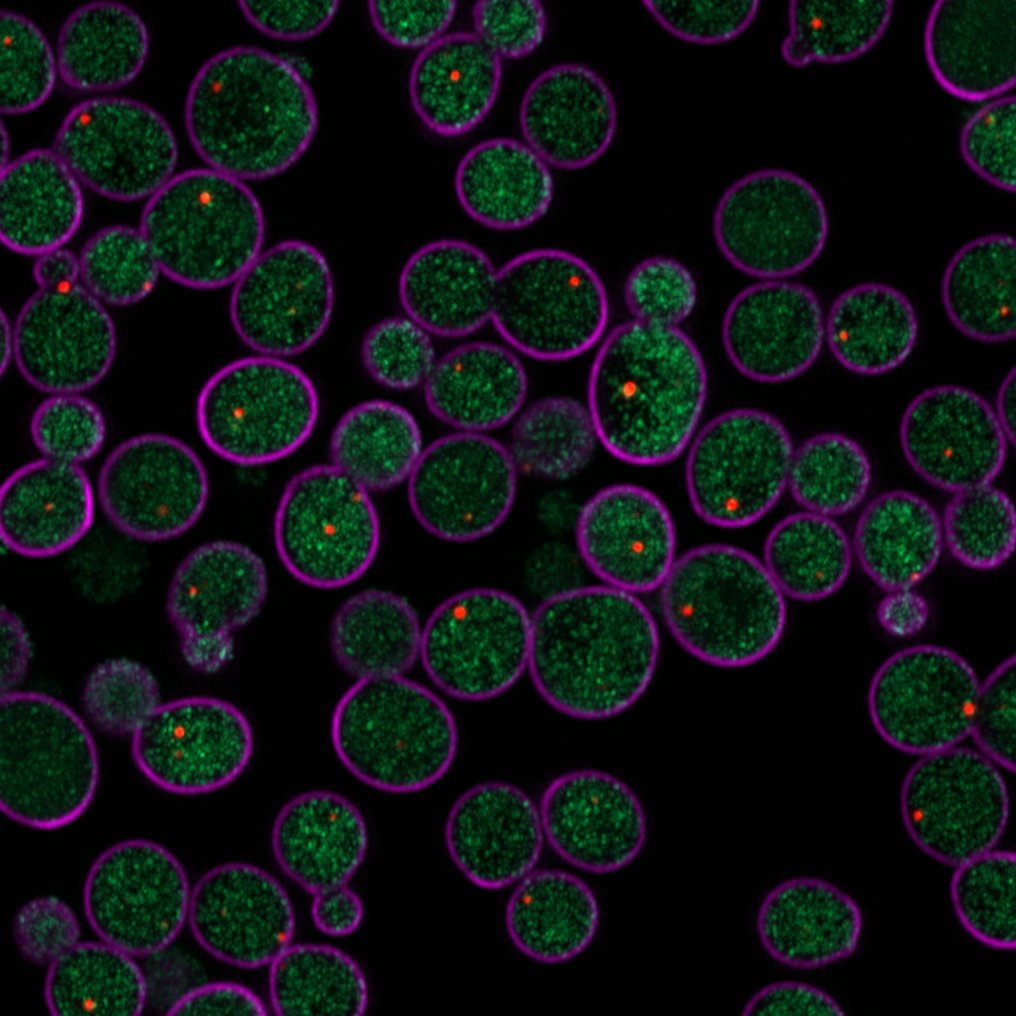

Supplement: Supplementary file 10 — Statistical source data. [file 41557_2024_1456_MOESM10_ESM.zip › Extended Dara Fig2a_top_middle_yKX372.jpg]

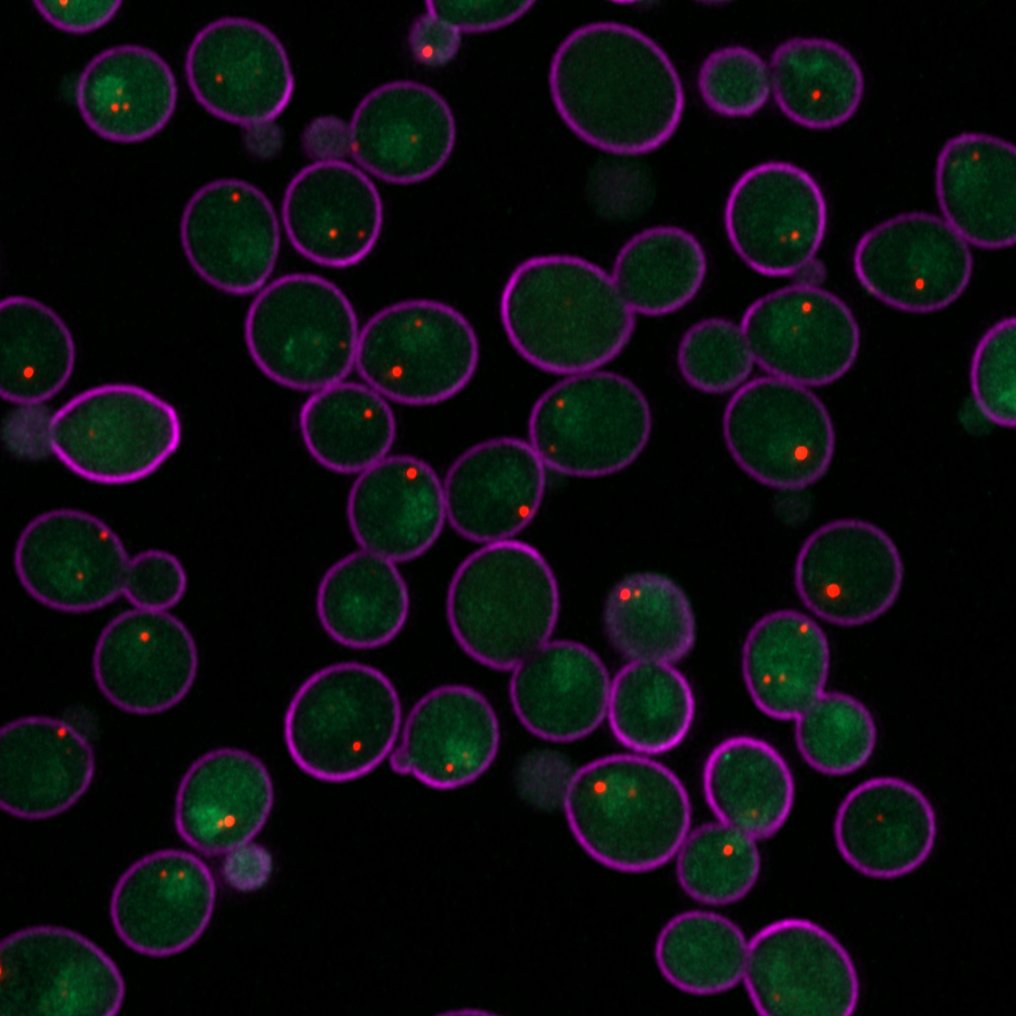

Supplement: Supplementary file 10 — Statistical source data. [file 41557_2024_1456_MOESM10_ESM.zip › Extended Dara Fig2a_top_right_yKX373.jpg]
